# Dietary cholesterol promotes repair of demyelinated lesions in the adult brain

**DOI:** 10.1038/ncomms14241

**Published:** 2017-01-24

**Authors:** Stefan A. Berghoff, Nina Gerndt, Jan Winchenbach, Sina K. Stumpf, Leon Hosang, Francesca Odoardi, Torben Ruhwedel, Carolin Böhler, Benoit Barrette, Ruth Stassart, David Liebetanz, Payam Dibaj, Wiebke Möbius, Julia M. Edgar, Gesine Saher

**Affiliations:** 1 Department of Neurogenetics, Max Planck Institute of Experimental Medicine, Hermann-Rein-Str. 3, 37075 Göttingen, Germany; 2 Institute of Neuroimmunology and Institute for Multiple Sclerosis Research, University Medical Centre Göttingen, Waldweg 33, 37073 Göttingen, Germany; 3 Department of Neuropathology, University Medical Center, Georg-August-University, Robert Koch Str. 40, 37075 Göttingen, Germany; 4 Department of Clinical Neurophysiology, Georg-August University, Robert Koch Str. 40, 37075 Göttingen, Germany; 5 Center Nanoscale Microscopy and Molecular Physiology of the Brain (CNMPB), Wilhelmsplatz 1, 37073 Göttingen, Germany; 6 Applied Neurobiology Group, Institute of Infection, Immunity and Inflammation, College of Medical Veterinary and Life Sciences, University of Glasgow, Glasgow G12-8TA, UK

## Abstract

Multiple Sclerosis (MS) is an inflammatory demyelinating disorder in which remyelination failure contributes to persistent disability. Cholesterol is rate-limiting for myelin biogenesis in the developing CNS; however, whether cholesterol insufficiency contributes to remyelination failure in MS, is unclear. Here, we show the relationship between cholesterol, myelination and neurological parameters in mouse models of demyelination and remyelination. In the cuprizone model, acute disease reduces serum cholesterol levels that can be restored by dietary cholesterol. Concomitant with blood-brain barrier impairment, supplemented cholesterol directly supports oligodendrocyte precursor proliferation and differentiation, and restores the balance of growth factors, creating a permissive environment for repair. This leads to attenuated axon damage, enhanced remyelination and improved motor learning. Remarkably, in experimental autoimmune encephalomyelitis, cholesterol supplementation does not exacerbate disease expression. These findings emphasize the safety of dietary cholesterol in inflammatory diseases and point to a previously unrecognized role of cholesterol in promoting repair after demyelinating episodes.

## Abstract (web summary)

Cholesterol is important for axonal myelination during development. Here the authors show that cholesterol levels are reduced in a cuprizone mouse model of multiple sclerosis and that dietary cholesterol supplementation enhances remyelination and recovery.

In demyelinating diseases such as multiple sclerosis (MS), the failure to remyelinate contributes to axonal damage^1^, a major factor in persistent disability. Remyelination failure can be attributed partially to an insufficient capacity of resident oligodendrocyte precursor cells (OPC) to proliferate, migrate, differentiate and initiate myelin membrane growth^2^,^3^. There is now good evidence to implement therapies that combine the established immunosuppressive treatment of MS with compounds that stimulate remyelination and hence may secondarily limit axonal damage^4^,^5^. A number of factors that support differentiation of OPCs have been reported recently, some of which are linked to cholesterol metabolism in differentiating oligodendrocytes^6^,^7^,^8^,^9^.

Patients with MS have disturbed brain lipid metabolism^10^, but serum lipid profiles are in the normal range^11^. During active disease and disease progression, total cholesterol levels can rise to the upper limit of the normal range^12^,^13^,^14^,^15^. Increased dietary intake of cholesterol is assumed to increase serum cholesterol and stimulate immunological responses in inflammatory diseases^16^. However, it is unclear whether the elevated serum cholesterol in MS patients (i) contributes to disease progression, (ii) is a consequence of acute disease or (iii) reflects an attempt to counterbalance the pathophysiological manifestation of the disease.

We previously showed that cholesterol is rate limiting for CNS myelination^17^ and that nutritional cholesterol supplementation can stimulate developmental myelination in a mouse model of leukodystrophy^18^. Here, we investigate the effects of increased dietary cholesterol on disease parameters in three distinct mouse models of MS, that is, on (i) inflammation and demyelination in experimental autoimmune encephalomyelitis (EAE), (ii) remyelination in lysolecithin induced lesions and (iii) demyelination and remyelination in the cuprizone model. High-cholesterol chow does not aggravate clinical symptoms nor inflammatory parameters in EAE or alter demyelination in cuprizone treated animals. Rather, we identify a novel function for cholesterol in myelin repair in adult mice. Dietary cholesterol modulates the profile of growth factors, simultaneously enhancing OPC proliferation and oligodendrocyte differentiation, thereby facilitating remyelination and reducing axonal injury. These data have implications for the treatment of demyelinating diseases.

## Results

### Cholesterol supplementation does not affect pathology in EAE

To test whether elevated serum cholesterol is a biomarker of acute inflammatory disease, we induced MOG-EAE and determined serum cholesterol at the peak of clinical symptoms, typically 16–20 days after induction. Surprisingly, in acute EAE, total serum cholesterol was reduced to about 75% of normal values (76±2 mg dl^−1^±s.e.m. cholesterol in EAE mice compared with 103±2 mg dl^−1^ in untreated controls, *n*=6–9, *P*<0.0001, Student's *t*-test). Similar reductions were observed during remission at 28 days after immunization (76±1 mg dl^−1^±s.e.m., *n*=18, *P*<0.0001 Student's *t*-test).

**Figure 1:**
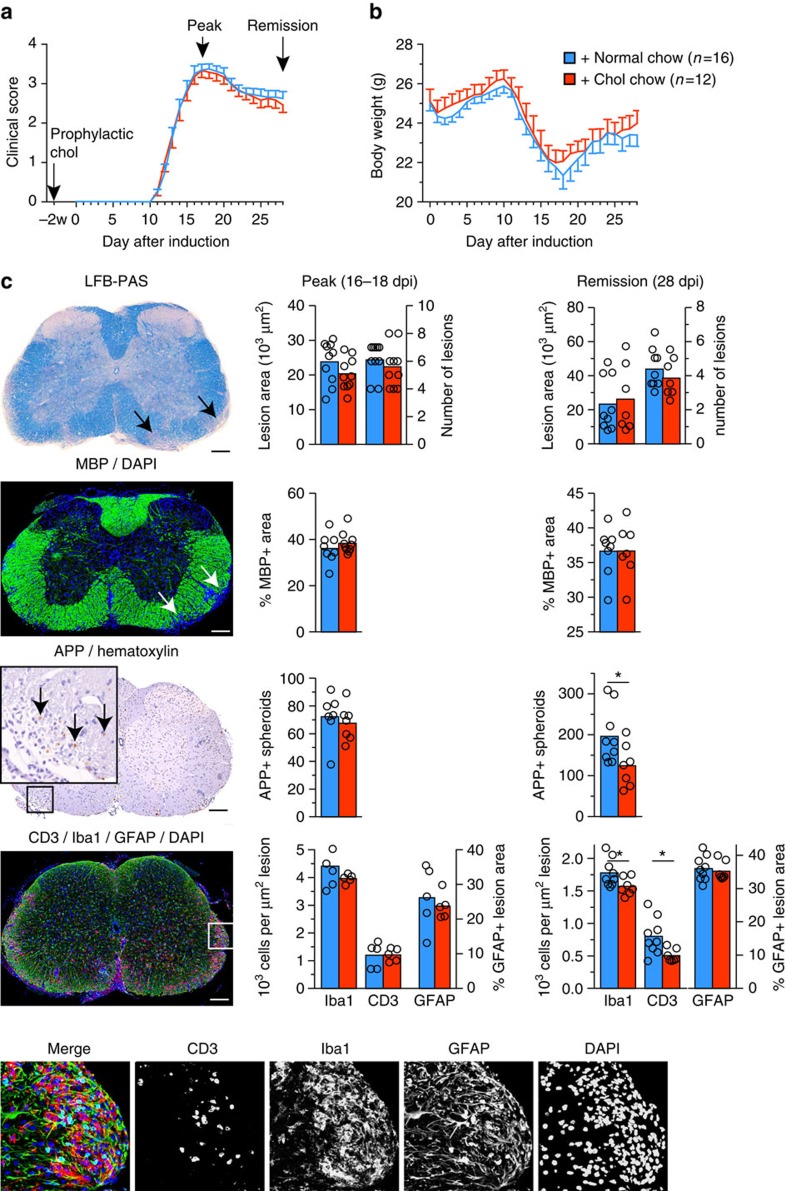
Dietary cholesterol does not aggravate EAE pathology. (**a**) Clinical score of mice with MOG-EAE on normal chow or chow supplemented with 5% cholesterol (*n*=12–16 mice, 2 independent experiments). Start of cholesterol feeding was prophylactic, two weeks before immunization. Arrows illustrate the time points of analyses at the peak of clinical symptoms (16–18 dpi) and at remission (28 dpi). (**b**) Body weight of experimental animals as in (**a**) assessed from the day of induction of EAE to the end of monitoring clinical scores (28 days). Data is expressed as mean weight±s.e.m. of *n*=12–16 animals. Onset of clinical symptoms was paralleled by a drop in body weight, and mice gained weight only after the peak of disease. (**c**) Lesion characteristics were determined on sections of lumbar spinal cord from mice fed normal chow or cholesterol enriched chow (*n*=5 animals, representative images on the left, scales 200 μm). Luxol fast blue-periodic acid-Schiff-hematoxylin (LFB/PAS) staining was used to determine the lesion area and number of lesions per section (arrow). Immuno-labeling for myelin basic protein (MBP) was used to determine the per cent of myelinated area within a lesion (defined in the DAPI channel as clusters of >20 nuclei, marked by arrows). On sections immuno-labeled for APP, the number of axonal speroids (arrows) per square mm white matter area was counted, as a readout of axonal damage. In remission, unpaired Student's *t*-test revealed significantly less axonal damage in cholesterol fed animals. Sections triple stained for microglia/macrophages, T cells, and astrocytes (Iba1-CD3-GFAP triple immuno-labeling) were used to assess the cellular composition of lesions. Unpaired *t*-tests revealed significantly reduced densities of microglia/macrophages and T cells in cholesterol fed animals (*, *P*<0.05). Bars represent mean values with individual data points.

Next, we asked whether dietary cholesterol supplementation worsens acute inflammatory disease. Unexpectedly, mice on a high-cholesterol chow (5% w/w cholesterol, fat content unchanged) either prophylactically, two weeks before inducing MOG-EAE, or therapeutically with onset of clinical symptoms, showed similar disease onset (normal chow 12.6±0.3 days; cholesterol 12.6±0.4d, *n*=12–16), mean clinical scores and body weight, as controls, during the 28 days of monitoring (Fig. 1a,b; Supplementary Fig. 1). Moreover, high-cholesterol chow did not correct the reduced serum cholesterol (77±8 mg dl^−1^, *n*=6). Correspondingly, at the peak of the clinical symptoms, dietary cholesterol did not influence the level of inflammation: histopathological lesions in the lumbar spinal cord white matter as well as the immune cell infiltration and characteristics of the pro-inflammatory milieu were comparable in extent and composition (Fig. 1c; Supplementary Fig. 2). These findings are in agreement with dietary cholesterol supplementation in the Theiler's virus model of MS (ref. 19). Nonetheless, inflammation was slightly ameliorated in cholesterol fed animals in remission, 28d after immunization (Fig. 1, Supplementary Fig. 2). Reduced infiltration of T cells and microglia/macrophages was accompanied by attenuated expression of several pro-inflammatory markers, such as interferon-γ (IFNγ), interleukin 17 (IL-17), granulocyte-macrophage colony-stimulating factor (GM-CSF), tumour necrosis factor (TNF), and major histocompatibility complex II (MHCII). Taken together, EAE is associated with decreased serum cholesterol that is not restored by supplemented cholesterol. Importantly, cholesterol does not exacerbate disease but even slightly ameliorates inflammation during remission, suggesting it is safe to administer in inflammatory diseases. As cholesterol supplementation promotes developmental myelination^18^, these data prompted us to examine cholesterol supplementation in a remyelination paradigm.

### Cuprizone lowers serum cholesterol and affects BBB integrity

We first tested whether serum cholesterol was altered in the cuprizone model of demyelinating disease (see also below). Surprisingly, after 4 weeks on cuprizone, mice had markedly reduced total serum cholesterol (76±3 mg dl^−1^±s.e.m. in comparison to 103±3 mg dl^−1^ in controls, *n*=9–13, *P*<0.0001, Student's *t*-test). Although liver function values were normal (Supplementary Fig. 3), we cannot exclude the possibility that this is due in part to altered liver metabolism^20^. In contrast to EAE, dietary supplementation with 2% w/w cholesterol normalized total serum cholesterol (106±5 mg dl^−1^, *n*=13).

**Figure 2:**
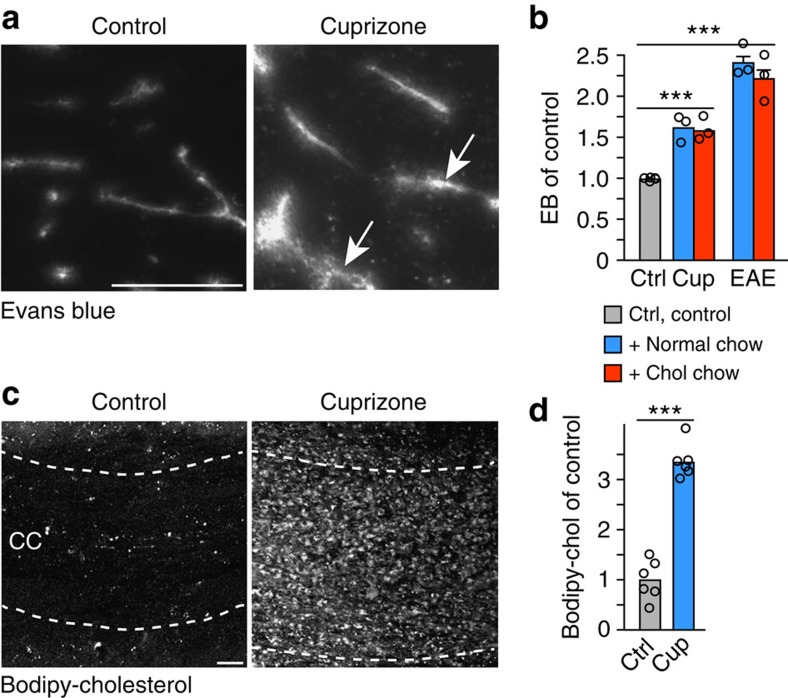
Increased BBB permeability in cuprizone treated mice. (**a**) Extravasation of Evans blue on sections of the corpus callosum. In control animals, Evans blue fluorescence is restricted to blood vessels but extravasates in mice on cuprizone (arrows) (scale, 50 μm). (**b**) BBB permeability was measured by Evans blue (EB) extravasation in brains of animals fed cuprizone (cup) for 5 weeks on normal chow or cholesterol supplemented chow, or in brains of animals with EAE 2d after the peak of clinical symptoms (*n*=3 animals). All treatment groups were normalized to untreated control animals (*n*=5) and compared by one way ANOVA (*P*<0.0001). Nutritional cholesterol did not influence BBB permeability. Bars represent mean±s.e.m. (**c**) Extravasation of bodipy-cholesterol. Maximum intensity projection of bodipy-cholesterol fluorescence in the corpus callosum (delineated by dashed lines) of mice that were kept on cuprizone for 5 weeks in comparison to untreated mice (control) (scale, 50 μm). (**d**) Quantification of bodipy-cholesterol extravasation after extraction. Data are expressed as fold changes±s.e.m. in cuprizone treated mice compared with untreated control animals (*n*=6 mice per group, unpaired Student's *t*-test, *P*<0.0001).

Under physiological conditions, the blood-brain barrier (BBB) prevents the passage of cholesterol from the circulation into the CNS (refs 21, 22). Therefore, we tested whether dietary cholesterol could penetrate the CNS in cuprizone fed mice. Surprisingly, BBB integrity was compromised in mice treated with cuprizone for 4 weeks, as indicated by extravasation of Evans blue dye into the CNS, following systemic administration (Fig. 2a,b). Systematic evaluation revealed increased BBB permeability during the entire treatment period of up to 12 weeks of cuprizone feeding (1.4±0.1 fold, *n*=4 P<0.05 Student's *t*-test).

The extent of extravasation was much smaller than in EAE, likely explaining why previous studies have missed this BBB defect^23^,^24^,^25^,^26^. Notably, dietary cholesterol did not influence BBB permeability. When tested one week after a single injection of bodipy-cholesterol, the fluorescence from this cholesterol derivative (its biophysical properties are very similar to unmodified cholesterol^27^) was readily detectable in the corpus callosum of cuprizone fed mice (in contrast to untreated controls) with a pattern typical for an intracellular localization, potentially in glial cells (Fig. 2c). Quantification of extravasated bodipy-cholesterol revealed a ∼3-fold increase in comparison to control mice (Fig. 2d). Thus, in cuprizone fed mice, peripheral cholesterol can cross the BBB.

### Cuprizone mediated demyelination is unaltered by cholesterol

**Figure 3:**
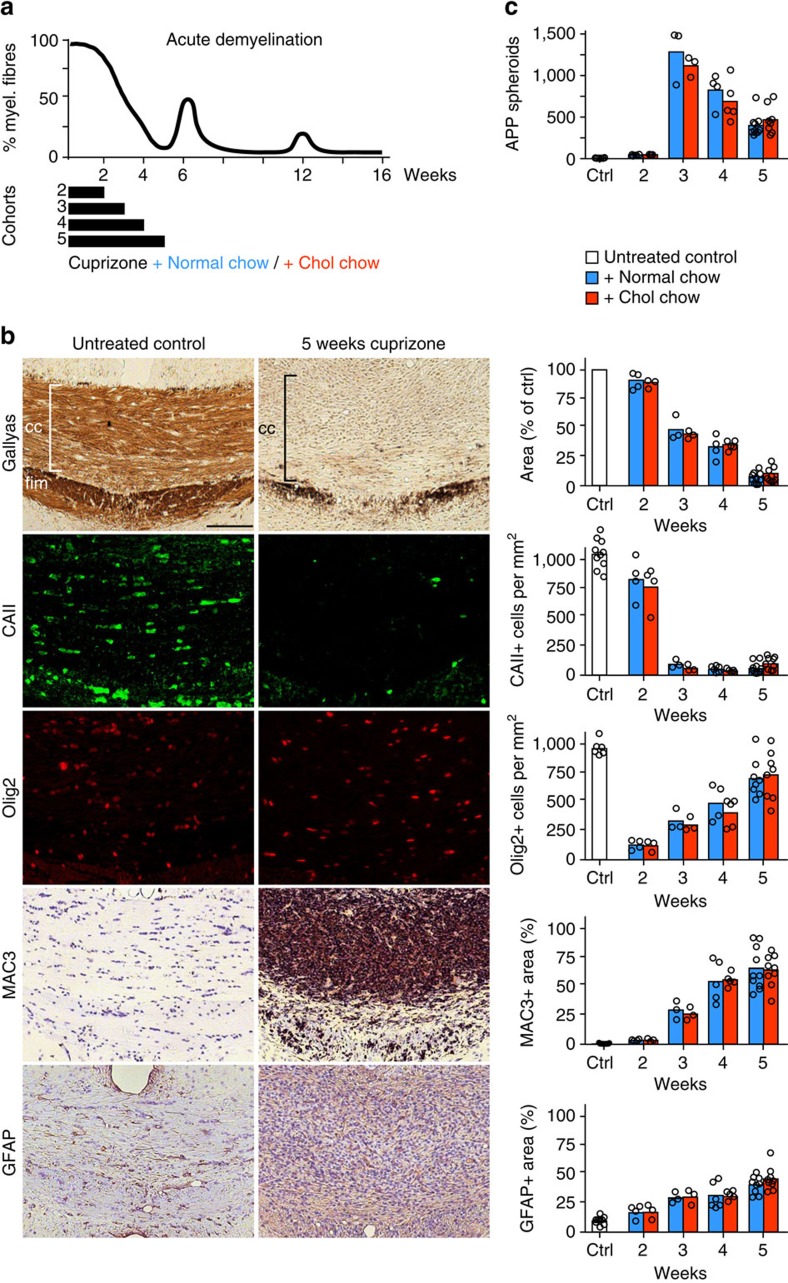
Cholesterol does not affect cuprizone mediated demyelination. (**a**) Scheme depicting the time course of demyelination/remyelination during 6 week cuprizone feeding (upper panel, based on own results and on other studies^25^,^28^) to the treatment paradigm. To assess the influence of high-cholesterol feeding on demyelination, mice on normal chow or high-cholesterol chow additionally received cuprizone in the diet for between 2 and 5 weeks (black bars) after which mice were analysed histologically. (**b**) Representative pictures of the corpus callosum of untreated control mice and mice after 5 weeks on cuprizone with the corresponding quantification on the right. Assessed were myelination (Gallyas silver impregnation), the number of mature oligodendrocytes (CAII), the number of oligodendrocyte lineage cells (Olig2), activated microglia (MAC3) and astrocytes (GFAP). Each bar represents the mean value for 3–5 (week 2–4) or 9–10 (week 5; untreated controls, ctrl) animals per condition with individual data points (scale 100 μm). (**c**) APP positive spheroids per mm^2^ in the corpus callosum at the end of 2–5 weeks of cuprizone with or without cholesterol supplementation (*n*=3–4 animals at 2 and 3 weeks, *n*=4–5 at week 4, *n*=6 untreated controls, *n*=9–10 at week 5).

Next we tested whether nutritional cholesterol altered histopathology during the demyelination phase of cuprizone treatment (Fig. 3a)^25^,^28^. In the corpus callosum, oligodendrocyte loss and demyelination evolved over the same time course in control and cholesterol supplemented mice (Fig. 3b), leading to almost complete depletion of mature oligodendrocytes after four weeks. In addition, oligodendroglial numbers (Olig2, oligodendrocyte lineage transcription factor 2 marks OPCs and oligodendrocytes), astrogliosis (GFAP, glial fibrillary acidic protein) and microgliosis (MAC3, macrophage-3 antigen) steadily increased in a comparable manner in both groups, and axonal damage (APP positive spheroids, Fig. 3c) was similar at all time points tested. Taken together, cholesterol supplementation does not interfere with the cuprizone treatment, and mature oligodendrocytes do not escape the toxic insult.

### Cholesterol facilitates remyelination and motor learning

**Figure 4:**
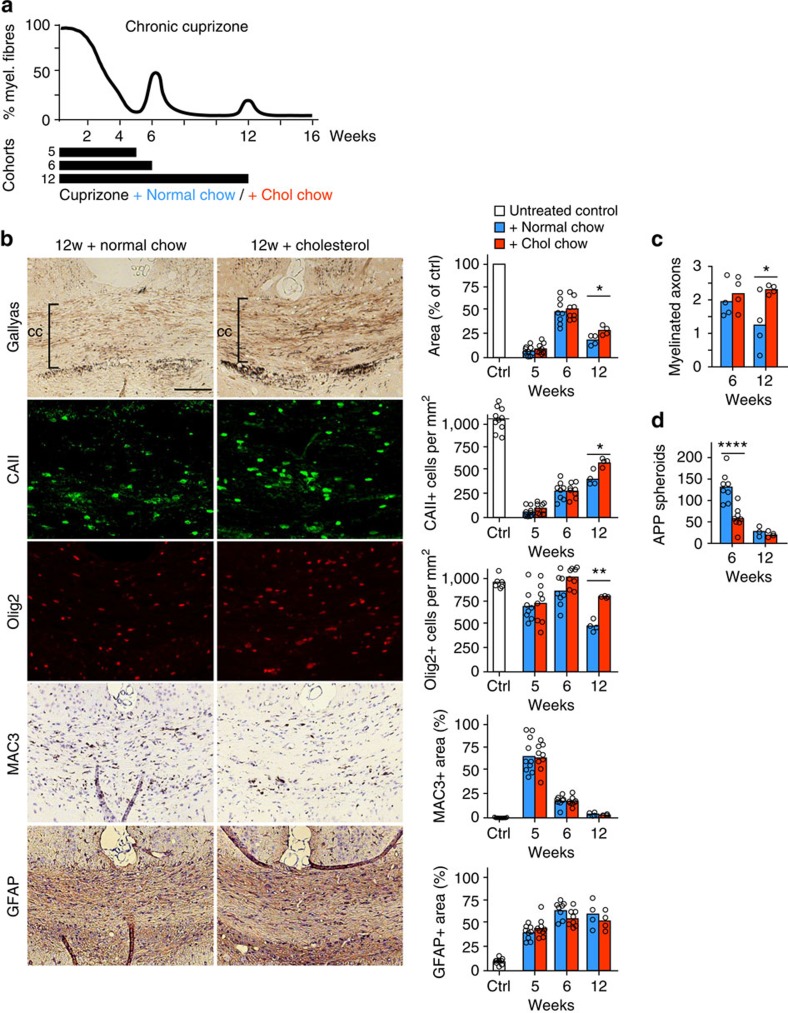
Cholesterol facilitates remyelination after chronic cuprizone exposure. (**a**) Scheme depicting the time course of demyelination/remyelination during cuprizone feeding to the treatment paradigm. To assess the influence of high-cholesterol feeding on spontaneous remyelination, mice received cuprizone in normal chow or chow supplemented with cholesterol for 5, 6 or 12 weeks (black bars) after which mice were analysed histologically. (**b**) Evaluation of disease in the corpus callosum of mice that were treated with cuprizone for 5, 6 or 12 weeks on normal chow or chow enriched with cholesterol. Corresponding representative pictures of the 12 weeks treatment cohort are on the left. Assessed were myelination (Gallyas silver impregnation), the number of mature oligodendrocytes (CAII), the number of oligodendrocyte lineage cells (Olig2), activated microglia (MAC3) and astrocytes (GFAP). Each bar represents the mean value of 4 (week 12) or 8–10 (week 5, 6) animals per condition with individual data points (scale 100 μm). (**c**) Myelinated axons per 10 μm^2^ in the corpus callosum at the end of 6 and 12 weeks of cuprizone with or without cholesterol supplementation (*n*=4 animals, Two-way ANOVA and Sidak's post test). (**d**) APP positive spheroids per mm^2^ in the corpus callosum at the end of 6 and 12 weeks of cuprizone with or without cholesterol supplementation (*n*=3–8 animals, Two-way ANOVA and Sidak's post test). Asterisks represent significant differences with *P<0.05; **P<0.01; ****P<0.0001.

Next, we tested the hypothesis that dietary cholesterol supplementation enhances adult remyelination. When mice are continuously exposed to cuprizone, an episode of spontaneous repair occurs in the sixth week, resulting in marked remyelination (Fig. 4a)^25^,^29^. At this time point, cholesterol neither influenced oligodendrocyte numbers, remyelination nor glial responses (Fig. 4b,c). However, the density of APP positive axonal spheroids in cholesterol fed animals was reduced, suggesting attenuated axonal damage (Fig. 4d).

After chronic cuprizone exposure (12 weeks), a second episode of weak and transient remyelination (up to 20% of full myelination) occurs (Fig. 4a). However, even if cuprizone is withdrawn at this point, repair is very limited^30^. Thus, despite a considerable density of OPCs and mature oligodendrocytes, remyelination is marginal and astrogliosis substantial (Fig. 4b, blue bars at 12 weeks). Remarkably, cholesterol supplementation increased remyelination ∼1.6-fold as assessed in Gallyas silver impregnated sections (Fig. 4b) and in electron micrographs of the corpus callosum (Fig. 4c, Supplementary Fig. 4). Coupled to this, a similar increase in OPCs and in mature oligodendrocytes was observed (Fig. 4b, 12 weeks). In addition, the positive influence of cholesterol was associated with increased body weight (Supplementary Fig. 5). Thus, in the context of recurrent depletion of mature oligodendrocytes, cholesterol supplementation enhances tissue repair.

**Figure 5:**
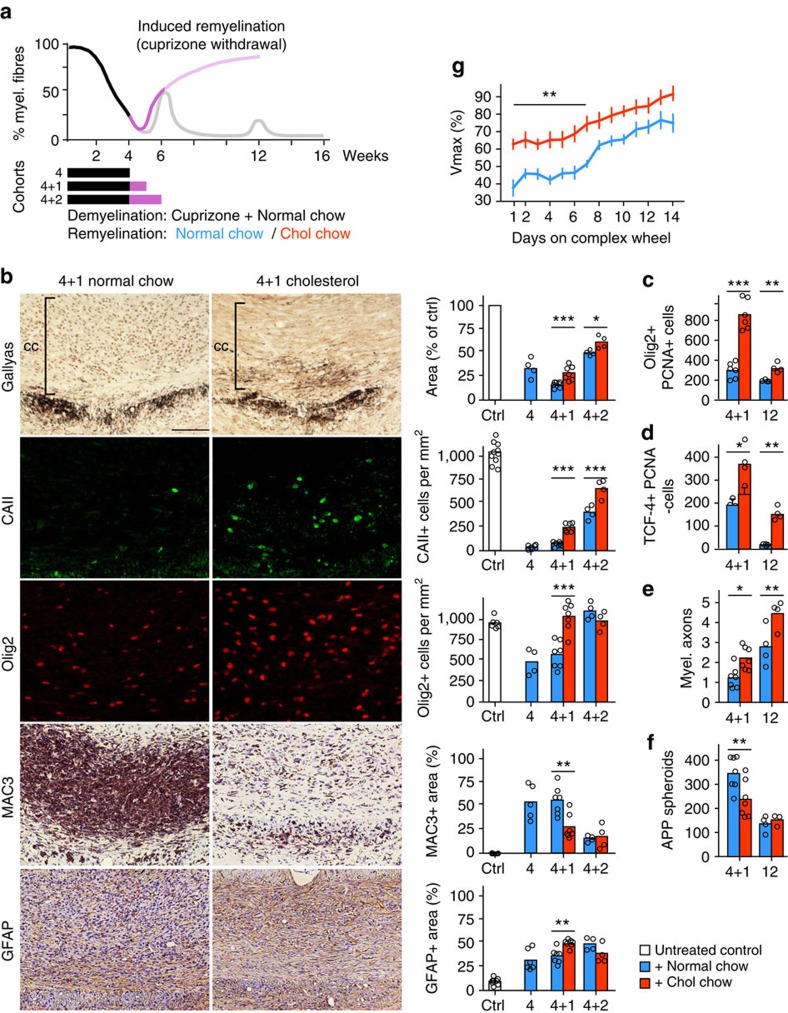
Cholesterol facilitates remyelination after cuprizone withdrawal. (**a**) Scheme depicting the time course of demyelination/remyelination during cuprizone feeding (remyelination after cuprizone withdrawal in purple). The influence of cholesterol on remyelination was assessed by feeding mice cuprizone in normal chow for 4 weeks (4, black bars) followed by ‘induced remyelination' after cuprizone withdrawal for 1 (4+1) or 2 (4+2) weeks on normal chow or cholesterol supplemented chow. (**b**) Representative pictures of the corpus callosum of mice after one week (4+1) remyelination. Corresponding quantification is on the right also including values for 2 weeks remyelination (4+2). Assessed were myelination (Gallyas silver impregnation), the number of mature oligodendrocytes (CAII), the number of oligodendrocyte lineage cells (Olig2), activated microglia (MAC3), and astrocytes (GFAP). Each bar represents the mean value from *n*=4–5 (4 and 4+2) or *n*=7 (4+1) animals (scale, 100 μm; Two-way ANOVA and Sidak's post test). (**c**) Quantification of proliferating OPCs (PCNA positive Olig2 positive) in the corpus callosum of mice after 4+1 treatment paradigm (4+1) or after 12 weeks (12) of cuprizone. Each bar represents the mean of *n*=6–7 (week 4+1), or *n*=4 (week 12) animals (Student's *t*-test). (**d**) Quantification of newly differentiated postmitotic oligodendrocytes (TCF4 positive, PCNA negative) in the corpus callosum treated as in **c**). Each bar represents the mean of *n*=6–7 (week 4+1), or *n*=4 (week 12) animals (Student's *t*-test). (**e**) Myelinated axons per 10 μm^2^ in the corpus callosum at the end of the 4+1 (*n*=7) and 4+2 (*n*=4) treatment paradigm (two-way ANOVA and Sidak's post test). (**f**) APP positive spheroids per mm^2^ in the corpus callosum (4+1 *n*=7; 4+2 *n*=3–4 animals, two-way ANOVA and Sidak's post test). (**g**) Motor learning as assessed by maximum velocity (Vmax) on a complex wheel (*n*=6 animals), expressed as per cent of the Vmax on a training wheel (mean of the last 7 days before changing to a complex wheel). Statistical evaluation of Vmax was done by Two-way ANOVA (cholesterol effect *P*<0.0001) and Sidak's post tests. Asterisks represent significant differences with *P<0.05; **P<0.01; ***P<0.001.

To specifically determine the effect of cholesterol during remyelination, we exposed mice to cuprizone for four weeks to achieve complete demyelination, then withdrew cuprizone to induce remyelination (‘induced remyelination') (Fig. 5a). Mice fed normal chow during the first 7 days after cuprizone withdrawal demyelinated further and had only slightly increased oligodendrocyte densities (Fig. 5b, compare blue bars 4 and 4+1). In contrast, cholesterol supplementation following cuprizone withdrawal dramatically increased OPC proliferation and augmented Olig2 positive cell density 1.5-fold (Fig. 5b,c). Densities of newly differentiated TCF4+ PCNA− (TCF4, also called TCF7L2, transcription factor 7-like 2; PCNA, proliferating cell nuclear antigen) oligodendrocytes were also increased by cholesterol (Fig. 5d, Supplementary Fig. 6), similarly as found in actively repairing lesions from patients with MS^31^,^32^,^33^. The resulting 2.7-fold increase in mature oligodendrocytes (Fig. 5b, time point 4+1) led to a 1.8-fold increase in myelin content on Gallyas silver impregnated sections and on electron micrographs (Fig. 5e; Supplementary Fig. 4). Cholesterol supplementation also altered the glial response, leading to a ∼30% increase in astrocytes and ∼50% reduction in microglial cells (Fig. 5b, 4+1). Axonal damage was attenuated to ∼70% in cholesterol fed animals of controls (Fig. 5f). These histological signs of repair were associated with a net gain in body weight, occurring within 7 days of cholesterol supplementation and contrasting with weight maintenance in mice fed normal chow (Supplementary Fig. 5). The beneficial effect of cholesterol persisted, leading to a robust increase in mature oligodendrocytes and myelin content at 2 weeks after cuprizone withdrawal (Fig. 5b, 4+2); a result that was confirmed on electron micrographs (Fig. 5e).

**Figure 6:**
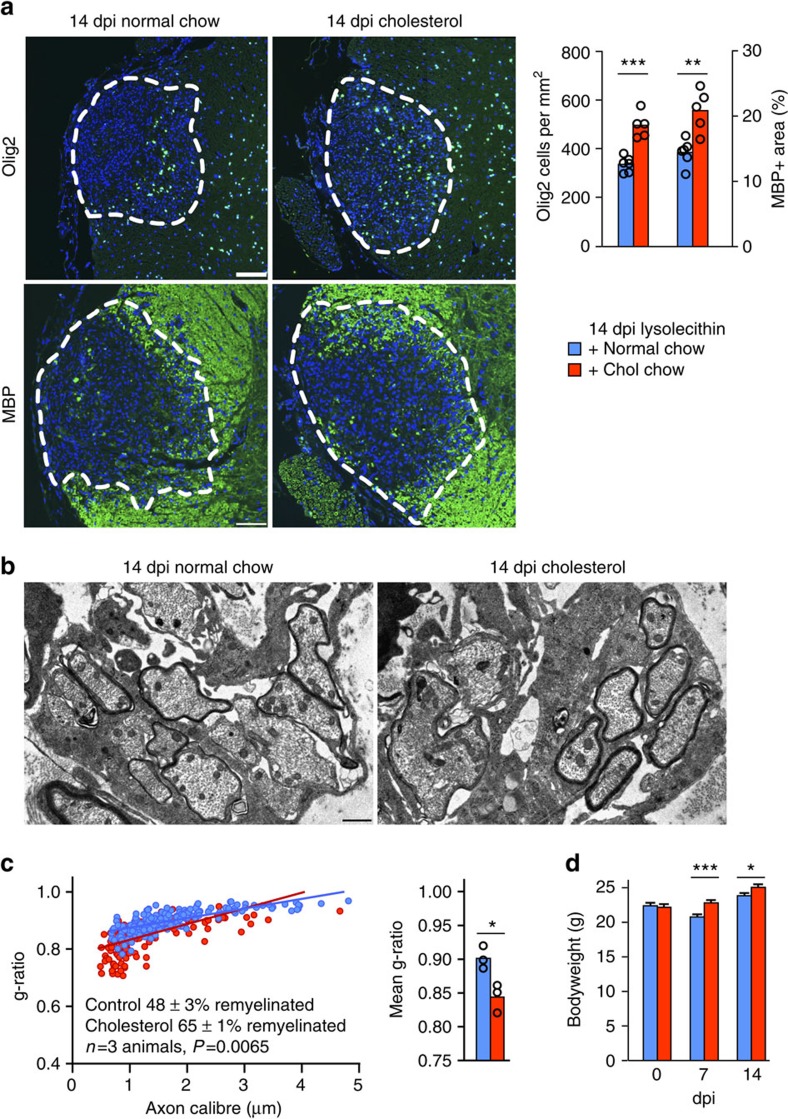
Cholesterol supports remyelination in the lysolecithin model. (**a**) Representative images of spinal cord sections 14 days post lesion (dpl) with 1 μl 1% lysolecithin in the ventral spinal cord with quantification of *n*=5 (cholesterol chow) and *n*=6 (normal chow) animals. Student's *t*-tests revealed significantly more Olig2 positive oligodendroglial cells within the lesion area (*P*<0.0001), and significantly more MBP positive area (*P*=0.027; scales, 100 μm). (**b**,**c**) Representative electron micrographs (scale 1 μm) and quantification of myelin sheath thickness and the portion of remyelinated axons in control and cholesterol fed mice at 14 dpl by g-ratio analysis (*n*=3 animals per group). (**d**) Body weight of experimental animals assessed at the day of lesion (day 0), after 7d and after 14d at the end of the experiment. Shown are the means±s.e.m. of *n*=9 (chol chow) to 10 (normal chow) animals. Two-way ANOVA with Sidaks post tests revealed a significant influence of cholesterol feeding at both time points (7 dpi *P*<0.0003, 14 dpi *P*=0.0362).

To examine the generality of this response, we investigated whether dietary cholesterol enhanced remyelination in another, completely distinct *in vivo* model of remyelination that is accompanied by confined BBB disruption. Localized injection of lysolecithin into the ventral-lateral spinal cord of adult mice was used to produce focal demyelination. As in the cuprizone model, demyelination was associated with a reduction in serum cholesterol to about 70% of untreated controls. Further, dietary cholesterol (2% w/w for 14 days) increased serum cholesterol slightly (79±3 mg dl^−1^±s.e.m. in cholesterol fed mice compared with 72±6 mg dl^−1^ in chow fed controls, *n*=3–5), enhanced remyelination and significantly increased the density of oligodendroglial cells within the lesion (Fig. 6a–c). The beneficial effect of cholesterol was also reflected in significantly increased body weight, relative to chow fed mice (Fig. 6d).

To investigate whether the histopathological improvements in cholesterol fed animals was associated with improved clinical measures, we returned to the ‘induced remyelination' paradigm in the cuprizone model (for a scheme of experimental paradigm, see Supplementary Fig. 7), measuring the maximum running velocity (Vmax) on a running wheel. First, a training wheel with regularly spaced rungs was placed into the cages to improve cardiopulmonary and musculoskeletal strength. One week after cuprizone withdrawal, the training wheel was replaced by a complex wheel with irregularly spaced rungs to measure bilateral sensorimotor coordination^34^ (Supplementary Fig. 7). The Vmax of mice remyelinated on normal chow dropped to about 40% of levels on the training wheel, and did not improve above 75% (Fig. 5g). In contrast, mice receiving cholesterol supplementation showed a less severe drop in Vmax (to 63%), followed by a steady increase that reached the velocity achieved on the training wheel after two weeks. Importantly, in control mice (without cuprizone) cholesterol supplementation, did neither influence performance on the training wheel nor motor learning (Vmax, run duration, number of runs and running distance on the complex wheel) (Supplementary Fig. 7 and not shown). Hence, cholesterol supplementation enhances repair after demyelination and improves neurological outcomes by supporting oligodendrocyte proliferation and differentiation, promoting remyelination, decreasing microgliosis, and attenuating axonal damage in a permissive environment (‘induced remyelination' after cuprizone withdrawal).

### Cholesterol changes the expression profile of growth factors

**Figure 7:**
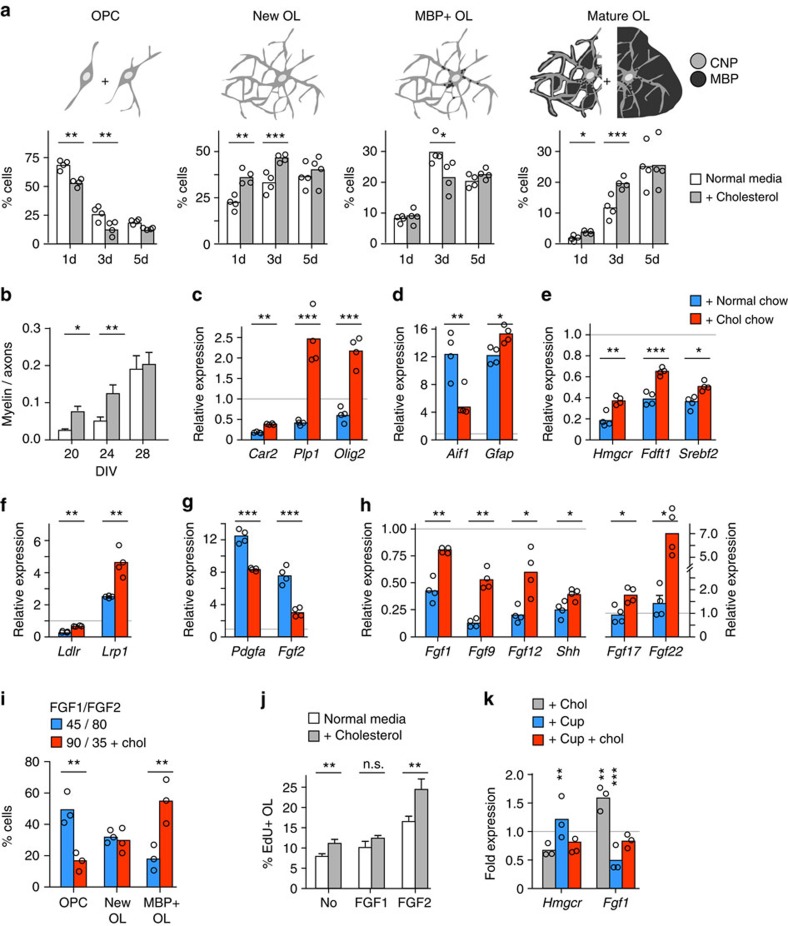
Cholesterol alters the expression profile of growth factors. (**a**) Differentiation time course of OPCs in oligodendroglial enriched cultures in the presence or absence of cholesterol supplementation (bars represent mean of *n*=4 cultures with individual data points). Drawings illustrate chosen categories of oligodendrocyte differentiation. In each category, significance was assessed by two-way ANOVA and Sidak's post tests. (**b**) Myelination at 20–28 days *in vitro* (DIV) in myelinating cocultures in the presence or absence of cholesterol (*n*=5–9 cultures). Myelin segments and axons were counted (see Supplementary Fig. 8b; two-way ANOVA and Sidak's post tests). (**c**–**h**) Quantitative RT-PCR analysis on dissected corpus callosi from mice after ‘induced remyelination' (4+1 weeks) and controls determining the expression of oligodendrocyte and myelin related genes (**c**; *Car2, Plp1, Olig2*), marker genes for microglia (*Aif1*) and astrocytes (*Gfap*) (**d**), genes involved in cholesterol synthesis (**e**; *Hmgcr, Fdft1, Srebf2*) and uptake (**f**; *Ldlr, Lrp1*), and growth factors downregulated (**g**; *Pdgfa, Fgf2*) and upregulated by cholesterol supplementation (**h**; *Fgf1, Fgf9, Fgf12, Shh, Fgf17, Fgf22*). Bars represent the means (*n*=4 animals) with individual data points (Student's t tests) normalized to untreated control mice (set to 1, grey line). (**i**) Differentiation of rat oligodendroglial cells in cultures supplemented with FGF1 and FGF2 (concentrations in ng per ml as indicated) in the presence or absence of cholesterol. Bars represent mean percentage of cells in each category of *n*=3 cultures (two-way ANOVA with Sidak's post test). (**j**) Proliferation of OPCs in response to growth factors and cholesterol. OPCs were cultured in the presence or absence of the growth factors (100 ng ml^−1^) FGF1 or FGF2 with or without cholesterol for 24 h. Data are mean EdU positive cells of all oligodendroglial cells±s.e.m. (*n*=13 (no GF, FGF2) or *n*=7 (FGF1) cultures of individual rats; Student's *t*-tests). (**k**) Quantitative RT-PCR on primary astrocytes treated with cuprizone (cup) with or without cholesterol (chol) supplementation. Bars represent the mean of *n*=3 independent experiments with individual data points compared with untreated cultures (set to 1, grey line; one-way ANOVA with Sidak's post tests. Asterisks represent significant differences with *P<0.05; **P<0.01; ***P<0.001.

To obtain insight into the mechanism by which cholesterol supports the simultaneous expansion of OPC and oligodendrocyte densities, we monitored differentiation of cultured primary oligodendrocytes in defined Sato media, with or without cholesterol supplementation. Oligodendrocytes differentiated significantly faster in the presence of cholesterol, as indicated by expression of differentiation markers and morphological changes (Fig. 7a, Supplementary Fig. 8a). However, the final stage of maturation after 5d in culture was unchanged, as shown previously^18^. Similarly, the rate of myelination as measured by MBP (myelin basic protein) positive area per axonal area (SMI31, phosphorylated axonal neurofilaments), was increased in spinal cord co-cultures differentiated in the presence of cholesterol (Fig. 7b, Supplementary Fig. 8b); neither the final degree of neurite outgrowth^35^ nor myelination, were influenced by cholesterol. These findings suggest that external cholesterol directly facilitates oligodendrocyte differentiation and the synthesis of myelin membranes.

In principle, a substantial induction of OPC differentiation could be unfavourable, if it occurs at the expense of OPC numbers. Indeed, gradual depletion of OPCs was observed in cholesterol supplemented oligodendroglial cultures (Fig. 7a, left panel). Thus, the expansion of proliferative OPCs *in vivo* (Fig. 5c) is likely an indirect consequence of additional factors from the local environment. To identify factors that mediate cholesterol dependent OPC proliferation, we analysed another cohort of mice in the ‘induced remyelination' treatment paradigm (4+1 weeks), using quantitative RT-PCR on dissected corpus callosi. In agreement with our histological data, oligodendrocyte related genes were (i) strongly downregulated in cuprizone fed mice in comparison to untreated controls (grey line) and (ii) significantly enhanced in cholesterol fed animals in comparison to chow fed animals (Fig. 7c, compare Fig. 5, Supplementary Table 1). Similarly, the astrogliosis (*Gfap*) and diminished microgliosis (*Aif1*, allograft inflammatory factor 1) were also reflected in the expression levels of respective marker genes (Fig. 7d). Surprisingly, cholesterol supplementation did not lead to feedback inhibition of cholesterol synthesis, but rather, increased the expression of genes involved in cholesterol synthesis and uptake (Fig. 7e,f), likely indicating enhanced remyelination. In contrast, expression of LXR family genes, which influence OPC differentiation^36^, was not affected by cholesterol (Supplementary Table 1).

The expression of growth factors involved in OPC survival, proliferation, migration or differentiation^28^, including *Igf1* (insulin-like growth factor), *Cntf* (ciliary neurotrophic factor), *Inhba* (inhibin beta-A, also called activin beta-A) and *Egf* (epidermal growth factor) was strongly increased (2–20 fold) by cuprizone, but was not further regulated by cholesterol supplementation (Supplementary Table 1). A set of genes whose products are known to inhibit differentiation of OPCs, such as *Fgf2* (fibroblast growth factor 2) and *Pdgfa* (platelet derived growth factor alpha)^37^,^38^,^39^, was also strongly upregulated by cuprizone (8–12 fold higher than untreated controls). Strikingly, the expression of these mitogens was attenuated in cholesterol fed animals to levels only 3–8 fold higher than in untreated controls (Fig. 7g). Moreover, in comparison to untreated controls, expression of another set of factors, some of which are known to facilitate differentiation of oligodendrocytes^39^,^40^, such as *Fgf1* and *Shh* (sonic hedgehog), was reduced by cuprizone, but strongly elevated by cholesterol supplementation (Fig. 7h). Expression of FGF receptors (1–3) was not influenced by cholesterol (data not shown). Demonstrating the generality of these findings (Supplementary Table 2), cholesterol influenced the profile of growth factor expression in a similar manner in mice treated chronically with cuprizone (12 weeks, see Fig. 4). In contrast to the ‘induced remyelination' paradigm, expression of enzymes involved in cholesterol synthesis was reduced in this cohort, suggesting feedback inhibition after remyelination is accomplished (Supplementary Table 2).

To determine whether the growth factor expression profile observed in cholesterol treated mice might be causally related to the enhanced repair, we tested whether these growth factor combinations directly enhance OPC differentiation *in vitro*, a surrogate for remyelination *in vivo*. Indeed, differentiation was enhanced when OPCs were cultured for 3 days in media supplemented with 90 ng ml^−1^ FGF1, 35 ng ml^−1^ FGF2 and cholesterol (exemplifying cuprizone+cholesterol chow), in comparison to 45 ng ml^−1^ FGF1 and 80 ng ml^−1^ FGF2 (exemplifying cuprizone+normal chow) (Fig. 7i). These data suggest the changes in growth factor expression are directly contributing to the improved repair.

Next, we cultured OPCs for 24 h in the presence of EdU (5-ethynyl-2′-deoxyuridine), a marker of cells in S-phase of the cell cycle, to determine whether the proliferative effect of growth factors was modified by cholesterol. Compared with vehicle treated controls, FGF2 doubled the number of EdU positive cells (as expected^41^), while FGF2 plus cholesterol elicited a threefold increase in this population (Fig. 7j), suggesting that cholesterol potentiates the effects of FGF2. Indeed, cholesterol alone only slightly increased the proportion of EdU+ cells in these cultures (Fig. 7j). We speculate that, despite attenuated *Fgf2* expression (compare Fig. 7g), potentiated FGF2 signalling contributes to the expansion of proliferating OPCs in cholesterol fed animals (compare Fig. 5c).

As only relatively few microglial cells are present in the corpus callosum of cholesterol fed mice in the ‘induced remyelination' paradigm (4+1 weeks) and in the ‘chronic cuprizone' paradigm (12 weeks), we hypothesized that astrocytes contributed principally to the altered profile of growth factors. Indeed, while primary astrocytes downregulated *Fgf1* expression in response to cuprizone, its expression was upregulated in response to cholesterol, irrespective of cuprizone (Fig. 7k), correlating with our *in vivo* data. Taken together, in the cuprizone model, cholesterol supplementation modulates the expression profile of growth factors, rebalancing proliferative and differentiation signals creating a permissive environment for repair.

## Discussion

Cholesterol availability is a prerequisite for myelination^17^,^42^,^43^ and, as we show here, exogenous cholesterol directly increases the rate of OPC differentiation. In agreement with our findings, failure to upregulate expression of sterol synthesis enzymes leads to arrested differentiation of *Tcf4* mutant OPCs, which can partially be rescued by cholesterol supplementation^44^. Further, cholesterol synthesis is enhanced during remyelination in mice^29^ and statin administration (inhibitors of sterol and isoprenoid synthesis) interferes with remyelination in the cuprizone model^45^. Nonetheless, monotherapy with statins ameliorates clinical scores in EAE; an effect associated with decreased CNS infiltration and inflammatory activity of T cells, likely reducing demyelination^46^,^47^,^48^. The outcomes of studies using statins in MS patients are contradictory, probably because of the disparate effects of statins on inflammation (beneficial^46^,^47^,^48^) and on remyelination (detrimental^45^). Accordingly, a recent meta-analysis does not recommend statin treatment for relapsing-remitting MS or clinically isolated syndrome^49^. Hence, we hypothesize that remyelination failure in MS reflects, at least partially, the inability to locally increase the cholesterol content in demyelinated lesions.

This hypothesis is supported by the current study. Exogenous cholesterol enters the CNS through an impaired blood-brain barrier, resulting in enhanced repair and an amelioration of the neurological phenotype in two distinct models of remyelination. Our data suggest that cholesterol directly facilitates repair by modulating the profile of growth factor expression, promoting OPC differentiation and, together with the mitogen FGF2, potentiating OPC proliferation. Importantly, cholesterol supplementation does not exacerbate inflammation in EAE.

What could prevent the increase of cholesterol in OPCs in demyelinated lesions? In patients with MS and in models of demyelination, CNS cholesterol homoeostasis is destabilized by a variety of mechanisms. First, expression of enzymes involved in cholesterol synthesis is reduced in demyelinated lesions (our study and refs 9, 29, 50). Second, intercellular cholesterol transport in the CNS is perturbed in patients with MS, because of reduced abundance of relevant proteins such as ApoE (Apolipoprotein E)^10^; and in mouse mutants with BBB disruption^22^, by uncontrolled flux of sterols in and out of the brain. BBB disruption has been shown by diffusion MRI in inflammatory diseases of the brain, such as MS (ref. 51) and, as we demonstrate here for the first time, is also a feature in the cuprizone model. Third, the decrease in serum cholesterol in both EAE and cuprizone mouse models, probably contributes to the impairment of CNS cholesterol homoeostasis. Whether patients with MS experience a drop in serum cholesterol during acute demyelinating episodes is unknown, and its analysis complicated by the fact that the standard first-line interferon beta treatment itself reduces total serum cholesterol^52^. Finally, the OPCs in chronically demyelinated lesions in MS^50^ and mouse models^29^, fail to upregulate lipid synthesis and differentiate, potentially as a consequence of an imbalance in signalling, as previously hypothesized^4^. Here, we demonstrate an imbalance in expression of growth factors in the cuprizone model, in accordance with previous studies^28^,^53^,^54^,^55^. The growth factor profile associated with cuprizone alone, such as high levels of FGF2 and PDGFa, is predicted to facilitate OPC proliferation but impede efficient remyelination, particularly after chronic demyelination.

Specifically, FGF signalling could critically influence the fate of demyelinated lesions^39^,^53^,^56^,^57^. FGF signalling comprises a very complex network, including 24 FGF family members and four different receptors, whose signalling outcome depends on various splice isoforms and on the multifaceted crosstalk between different pathways^58^. Here, we focused on the two major FGF members involved in myelination, FGF1 and FGF2. FGF2 has been implicated in OPC proliferation, migration and inhibition of oligodendrocyte differentiation^38^,^39^,^41^. In the cuprizone model and in patients with MS, FGF2 abundance correlates with the degree of OPC proliferation^28^,^53^,^56^,^59^. FGF2 is increased in regions of active OPC proliferation and ongoing remyelination, such as active lesions or the rim of demyelinated lesions, while it is downregulated in remyelinated shadow plaques, and it is low in abundance in normal appearing white matter and in the core of demyelinated silent lesions^59^. We show that cholesterol administration attenuates the overexpression of *Fgf2* and other mitogens in the cuprizone model. Surprisingly, this did not restrict proliferation but augmented OPC numbers, likely through synergy with cholesterol (see below).

FGF1 is reduced in active MS lesions^60^, and increased expression is only found in remyelinated lesions^56^. In contrast to FGF2, FGF1 is not mitogenic for OPCs (our study and refs 39, 56). Rather, FGF1 accelerates myelination *in vitro*^56^, to an extent remarkably similar to what we observed in cholesterol treated cultures. FGF1 might support CNS repair by inducing lipid synthesis and secretion by astrocytes^61^. The crosstalk between FGF signalling and regulation of cholesterol metabolism is supported by the presence of sterol responsive elements (consensus sequences for the SREBF2 transcription factor that increases cholesterol synthesis) in the *Fgf1* and *Fgf2* promoters (https://www.genomatix.de/). Altered cellular cholesterol levels can modulate signalling pathways, as shown for wnt signalling; the cholesterol content regulates the recruitment of different sets of scaffolding/adaptor proteins to the plasma membrane^62^. We demonstrate that cholesterol induces the expression of *Fgf1* in astrocytes; however, the affected signalling remains enigmatic. In addition to astrocytes, other cells such as microglia, OPCs, neurons and vascular cells likely participate in growth factor synthesis. In cholesterol treated mice, the expression profile of growth factors was altered such that the mitogens FGF2 and PDGFα were attenuated and differentiating cues such as FGF1 and Shh were enhanced. Other factors that are unaffected by cholesterol probably also contributed to the repair process.

**Figure 8:**
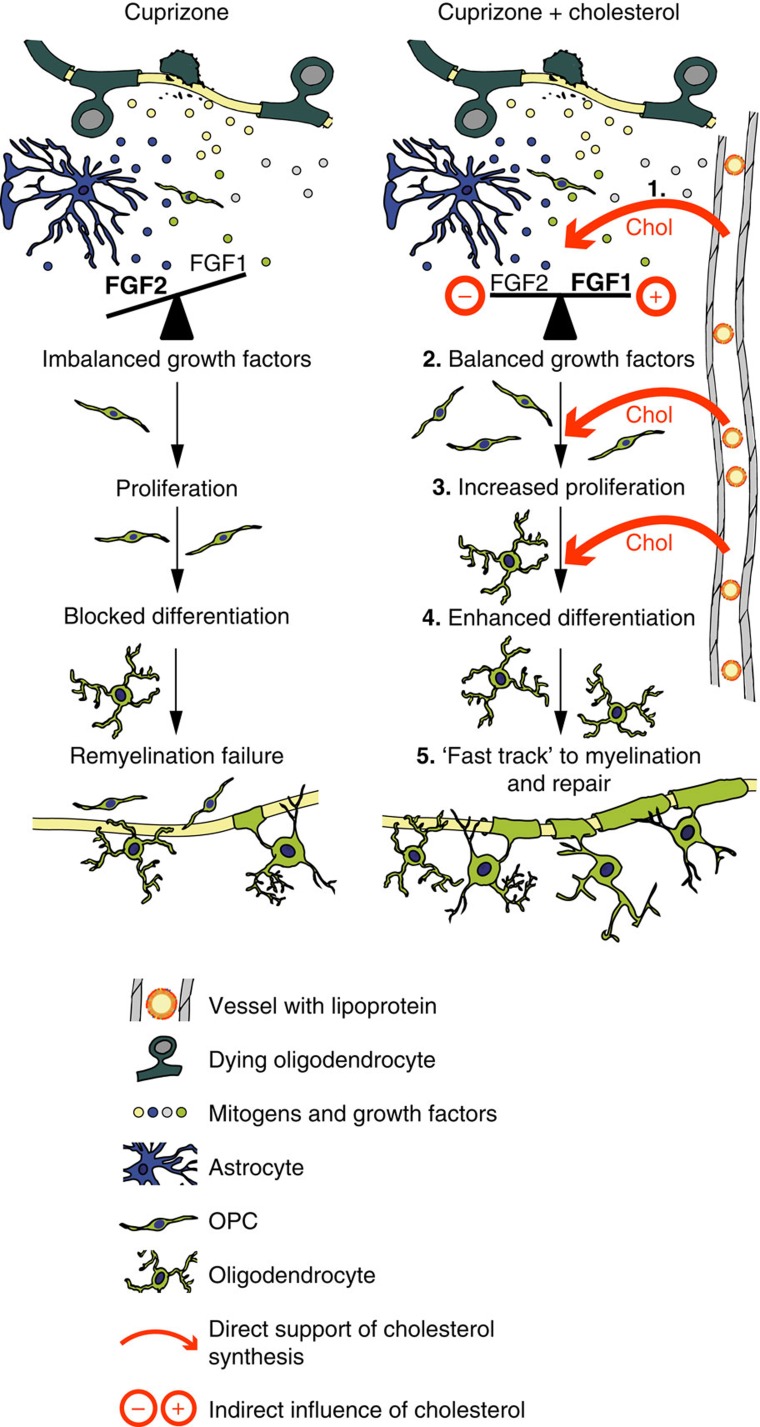
Working model of repair processes influenced by cholesterol. Working model of nutritional cholesterol mediated repair processes. Cuprizone exposure causes oligodendrocyte loss and demyelination and slow repair (left panel) because of OPC depletion, imbalanced growth factors, and low local availability of cholesterol. In case of nutritional supplementation, cholesterol from the circulation enters the CNS because of increased BBB permeability (red arrows) increasing the local cholesterol availability (1). There, cholesterol rebalances the expression of growth factors and mitogens synthesized e.g. by astrocytes (2). This simultaneously enhances OPC proliferation (3) and opens a window for OPC differentiation. Cholesterol directly facilitates oligodendrocyte differentiation, presumably by relieving cells from time and energy intensive cholesterol synthesis (4). Altogether, these effects provide a ‘fast track' to remyelination and repair (5).

Our data reveal a previously unknown function of nutritional cholesterol in adult remyelination (Fig. 8 shows a working model). In response to cuprizone-mediated demyelination in mice, the secreted mitogens and growth factors favour the proliferation and oppose the differentiation of OPCs, which slows and ultimately impairs remyelination. Dietary supplementation increases cholesterol availability within the demyelinated CNS and this is associated with rebalancing of growth factor expression. The altered profile of growth factors in cholesterol treated mice simultaneously facilitates OPC proliferation and oligodendrocyte differentiation *in vivo*. Thus arrested repair can be overcome by increasing the local availability of cholesterol which we achieved by nutritional supplementation.

We envision that moderate concentrations of the mitogen FGF2, when in synergy with cholesterol, potentiate OPC proliferation, and at the same time opens a window for OPC differentiation that is also enhanced by increased levels of pro-differentiation factors. In addition, cholesterol might directly facilitate oligodendrocyte differentiation, by relieving cells of the burden of establishing the complex time- and energy-intensive anabolic cholesterol pathway. Supplemented cholesterol can directly support myelination by incorporation into myelin membranes, as shown previously in a leukodystrophy model^18^. The current study suggests that cholesterol provides a ‘fast track' to remyelination and repair.

In contrast to the beneficial effect on remyelination, high-cholesterol chow (2% cholesterol) has no effect on demyelination and oligodendrocyte survival in the cuprizone model, likely because oligodendrocyte loss is induced by direct cuprizone-mediated damage, and is concomitant with a low-grade inflammatory cascade involving T cells, astrocytes and microglia^63^. Consistent with cholesterol supplementation not exacerbating demyelination in the cuprizone model, high-cholesterol chow (5% cholesterol) did not aggravate disease in EAE. Moreover, cholesterol supplementation attenuated axonal damage in both models; during active remyelination in the cuprizone model and during remission in EAE. Likely, this is secondary to ameliorated disease states and balanced expression of growth factors and pro-inflammatory factors. We found that dietary cholesterol slightly ameliorated inflammation in EAE, while, in contrast, a high-fat chow aggravates EAE symptoms^64^,^65^. Medium and long chain fatty acids of the high-fat chow probably modulate T cell differentiation^66^. Whether fatty acids contribute to the increased disease activity in some MS patients with elevated serum cholesterol remains unclear^12^. Importantly, feeding cholesterol appears to be safe in mice with inflammatory disease.

Taken together, our data show that demyelinating disease destabilizes peripheral and CNS cholesterol homoeostasis. Dietary cholesterol supplementation supports cholesterol metabolism in the CNS and has the remarkable potential to ameliorate disease by facilitating several repair mechanisms, leading to improved remyelination and neurological outcome. This study highlights the safety of dietary cholesterol and might have implications for the management of demyelinating diseases, but further studies, especially in combination with immune suppressive drugs, are required to determine its feasibility for patients.

## Methods

### Mice

All animal studies were performed in compliance with the animal policies of the Max Planck Institute of Experimental Medicine, and were approved by the German Federal State of Lower Saxony. Adult male C57BL/6N mice (8–10 weeks of age) were taken for all analyses. Animals were randomly assigned to an experimental group. Mice were fed normal chow (V1124 ssniff Spezialdiäten GmbH, Germany) or chow supplemented with either 2% w/w (cuprizone and lysolecithin experiments) or 5% w/w cholesterol (EAE experiments).

For MOG-EAE, mice purchased from Charles River were immunized subcutaneously with 200 μg myelin oligodendrocyte glycoprotein peptide 35–55 (MOG35–55) in complete Freund's adjuvant (*M. tuberculosis* at 3.75 mg ml^−1^) and i.p. injected twice with 500 ng pertussis toxin as described^67^. Animals were examined daily and scored for clinical signs of the disease. If disease did not start within 15 days after induction or the clinical score rose above 4, animals were excluded from the analysis. The clinical score was: 0 normal; 0.5 loss of tail tip tone; 1 loss of tail tone; 1.5 ataxia, mild walking deficits (slip off the grid); 2 mild hind limb weakness, severe gait ataxia, twist of the tail causes rotation of the whole body; 2.5 moderate hind limb weakness, cannot grip the grid with hind paw, but able to stay on a upright tilted grid; 3 mild paraparesis, falls down from a upright tiled grid; 3.5 paraparesis of hind limbs (legs strongly affected, but move clearly); 4 paralysis of hind limbs, weakness in forelimbs; 4.5 forelimbs paralyzed; 5 moribund/dead. Mice received 5% cholesterol chow commencing either two weeks before immunization defined as prophylactic regimen or at the first appearance of EAE symptoms defined as therapeutic regimen and continued until day 28.

For cuprizone experiments, mice were fed 0.2% w/w cuprizone (Sigma-Aldrich Inc., Germany) in powder chow with or without cholesterol for ‘demyelination' (2–5 weeks) and ‘chronic cuprizone' (6 and 12 weeks) paradigms. For ‘induced remyelination' experiments, mice were fed cuprizone in standard chow for 4 weeks, followed by cuprizone withdrawal and feeding mice standard chow with or without cholesterol supplementation. Mice were fed three times a week an exceeding amount of chow by dispenser. Food intake and animal weight was monitored. Age-matched untreated controls were fed standard powder chow.

Focal spinal cord demyelinating lesions were induced under anaesthesia by stereotactic injection of 1 μl lysolecithin (1%, from egg yolk, alpha-lysophosphatidylcholine, Sigma) into the ventro-lateral funiculus at Th10 of 8-week old animals, as previously described^68^. The injection was performed with a 10 μl Hamilton syringe, fitted with a thin tapered glass tip, at a rate of ∼1 μl min^−1^. This procedure created fusiform demyelinating lesions, 5–6 mm in length. At the day of injection, mice were randomly assigned to normal or 2% cholesterol chow for 14 days, after which the animals were killed, and the spinal cord processed for histology.

Bodipy-cholesterol injections were done as described^18^. Briefly, bodipy-cholesterol (Topflour, Avanti Polar Lipids) was injected i.p. (16 μg g^−1^ body weight). After one week, mice were perfused, and bodipy-cholesterol fluorescence was analysed on vibratome sections using a custom made two-photon laser scanning microscope equipped with a titanium-sapphire laser and a × 20 water immersion objective (NA 1.0). Z-stacks of 100-μm depth were obtained and processed to maximum intensity projections. For tracer quantification of bodipy-cholesterol (5 μg g^−1^ body weight, i.p. injection 7d circulation time) or Evans blue (50 μg g^−1^ body weight, i.v. injection, 4 h circulation time) animals were perfused with PBS to remove tracer from the circulatory system. Brains were dissected and immediately frozen on dry ice, weighed and stored at −80 °C for further processing. Tissue was lyophilized (Christ LMC-1 BETA 1-16) at –36 °C for 24 h under vacuum of 0.2 mBar. For tracer extraction, hemispheres were incubated shaking in 10 μl formamide per mg brain at 57 °C for 24 h. Integrated density of tracer fluorescence was determined in triplicates on a fluorescent microscope (Observer Z2, Zeiss, Germany), equipped with an AxioCam MRc3, × 1 Camera Adaptor and the ZEN 2012 blue edition software recorded at × 10 magnification (Plan-Apochromat × 10/0.45 M27). Tracer concentration was calculated using a standard curve and normalized to matched controls (set to 1).

Motor skill performance was assessed essentially as described^34^. Mice were randomly divided into two treatment and two control groups (*n*=6–13) and housed in individual cages that allow computer-controlled recording of wheel rotation as a function of time (MatLab-based custom software). The axis of each wheel was attached to a rotation sensor with a resolution of 16 per turn. One wheel revolution comes up to a running distance of 35.5 cm. The running wheel revolutions were recorded continuously at a sampling rate of 1/0.48 s by a customized recording device and software (Boenig & Kallenbach oHG, Dortmund, Germany). Mice in treatment groups were treated as in the ‘induced remyelination' paradigm (feeding 4 weeks cuprizone in normal chow followed by withdrawal of cuprizone and feeding normal chow or cholesterol supplemented chow). Control animals received normal chow for the entire experiment or were switched to cholesterol chow after 4 weeks. One week after the start of the experiment training wheels with regularly spaced rungs were placed into the cages for adaptation of cardiopulmonary and musculoskeletal strength. One week after the switch of diets (week 5 of experiment), wheels were replaced by complex wheels with irregularly spaced rungs to assess the bilateral sensorimotor coordination that likely involves the cerebellum and motor cortex and connecting white matter such as the corpus callosum. Specifically, we measured maximum running velocity (V_max_), in addition to the total maximum run duration (D_max_), accumulative distance in metres (Dist_ac_) and the number of individual runs (N_run_). Parameters were logged once daily (12 am).

### Serum analyses

Animals were fasted for 4 h, blood was collected from the retroorbital sinus, and serum was prepared after clotting by centrifugation. Cholesterol measurements were done with the architectII system (Abbott Diagnostics).

### Cell isolation and flow cytometry

Single-cell suspensions from spinal cords were obtained via mechanical dissociation on a cell strainer. Immune cells were separated over a two-phase Percoll-density gradient. Staining of αβTCR/CD4^+^ T cells, αβTCR/CD8^+^ T cells and CD45/CD11b cells (macrophages/microglia) was performed using the following antibodies in a 1:200 dilution: Anti-CD3e (clone 145-2C11), BioLegend; anti-CD4 (clone GK 1.5), BD; anti-CD8 (clone 53-6.7), BD; anti-CD8 (clone 53–6.7), BD; anti-CD11b (clone M1/70), BioLegend; anti-CD45.2 (clone 104), BioLegend. The addition of Calibrite APC beads (BD) allowed for cell quantification. Flow cytometry was performed using a FACSCalibur operated by Cell Quest software (Becton Dickinson).

### Histochemistry

Anesthetized mice were perfused with 4% formaldehyde (PFA). Brain samples of cuprizone treated animals were cut at Bregma 1.58 for comparable pathology because the extent of cuprizone mediated demyelination strongly depends on the rostral/caudal position^69^. Tissue was postfixed overnight, embedded in paraffin and cut into 5 μm sections (HMP 110, MICROM). Gallyas silver impregnation was done as described^18^. For immunohistological analyses, sections were deparaffinized followed by antigen-retrieval in sodium citrate buffer (0.01 M, pH 6.0). For immunofluorescence, sections were blocked with serum free protein block (Dako). Primary antibodies were diluted in 2% bovine serum albumin (BSA)/PBS and incubated for 48 h followed by fluorophor coupled secondary antibodies. For immunohistochemistry, endogenous peroxidase activity was blocked with 3% hydrogen peroxide. Sections were then blocked (20% goat serum in BSA/PBS) and incubated with primary antibodies. Detection was done with the LSAB2 kit (Dako, Hamburg, Germany) or the Vector Elite ABC kit (Vector Labs). HRP substrate 3,3′-Diaminobenzidine (DAB) was applied by using the DAB Zytomed Kit (Zytomed Systems GmbH). Haematoxylin stain was done to label nuclei. Sections were dehydrated before mounting (Eukitt). Specimens were analysed on an Axio Imager.Z1 (Zeiss) equipped with an AxioCam MRc3, × 0.63 Camera Adaptor and the ZEN 2012 blue edition software using × 10 objective (Plan Apochromat × 10/0.45 M27) or × 20 objective (Plan-Apochromat × 20/0.8) and evaluated with Image J software. Quantification of areas (Gallyas, GFAP, MAC3) were done by applying semi-automated ImageJ software macro to threshold (variable threshold in case of Gallyas and fixed threshold for antibody stainings) and colour deconvolute the images of the corpus callosum above the fornix (Bregma 1.58). Three to five sections per animal were analysed. Quantification of EAE lumbar spinal cord lesions was done on two to four quadruple stained sections (Iba1 (induction of brown adipocytes 1), CD3, GFAP, DAPI) per animal recorded with tile region setup and shading correction. Lesion area was defined by focal accumulation of at least 20 DAPI positive cells, the presence of microglia and infiltration of CD3 positive cells. Lesion area, number of Iba1 and CD3 positive cells and GFAP positive area were evaluated. Quantification of cuprizone treated animals: cell number (CAII (carbonic anhydrase 2), Olig-2, TCF4, PCNA), APP positive spheroids and area (Gallyas, GFAP, MAC3) was done in the corpus callosum above the fornix (Bregma 1.58). Three to five sections per animal were analysed. Microscope settings are listed in Supplementary Table 4.

Electron microscopic analysis was done as previously described^17^. Briefly, tissue was fixed in 4% PFA, 2.5% Glutaraldehyde, 0.1 M Phosphate buffer and sagittal sections were cut on a vibratome (Leica VT1200, 300 μm). The corpus callosum with adjacent tissue (−0.04 mm lateral) was punched with a 2 mm diameter punching tool and embedded in epon (EMTP, Leica). At least 15 digital pictures (× 12,000 magnification, TRS, Moorenweis) of uranyl acetate contrasted ultrathin sections were taken with the Zeiss EM900.

### Antibodies

The following antibodies were used: APP (Chemicon MAB348), CAII (Said Ghandour); CD3 (Serotec MCA1477);CD3e (Biolegend clone 145-2C11), CD4 (Becton Dickinson clone GK1.5), CD8 (Becton Dickinson clone 53-6.7), CD11b (Biolegend clone M1/70), CD45.2 (Biolegend clone 104), CNP (2′,3′-Cyclic-nucleotide 3′-phosphodiesterase, Sigma C5922), GFAP (Chemicon MAB3402), Iba1 (Wako 019-19741), MAC3 (Pharmigen 01781D); MBP (Serotec MCA409S), Olig2 (Prof Charles Stiles/ Dr. John Alberta, DF308), PCNA (Abcam ab29), SMI31 (Covance SMI-31P), TCF4 (Millipore 04-1080).

### Expression analyses

For the characterization of the proinflammatory milieu in EAE mice, RNA from total spinal cord lysates was isolated using Trizol (Thermo Fisher). complementary DNA (cDNA) was synthesized using RevertAid First Strand cDNA Synthesis Kit (Thermo Fisher) according to the manufacturer's protocol. Quantitative RT-PCR was performed using a StepOnePlus Real-Time PCR System operated by StepOnePlus Software v2.0. Target-specific FAM- and TAMRA-labeled TaqMan probes were used in all cases. Measurements were performed in independent duplicates. Gene expression was normalized to β-actin. Relative changes in gene expression were analysed via the 2ΔΔ*C*(T) method.

For expression analyses on brain sections, mice were killed by cervical dislocation and brains were quickly cooled and sliced coronally using a brain matrix (Asi-Instruments). The corpus callosum was dissected from Bregma +1.10 to −2.46 and RNA was extracted using RNeasy Mini (Qiagen). The concentration and quality of RNA was evaluated using a NanoDrop spectrophotometer and RNA Nano (Agilent). cDNA was synthesized with Superscript III (Invitrogen) and quantitative PCRs were done in triplicates with the GoTaq master mix (Promega) on a 7500 Fast Real-Time PCR System (Applied Biosystems). Expression values were normalized to the geometric mean of two housekeeping genes, Hprt (Hypoxanthin-Phosphoribosyl-Transferase 1) and Rplp0 (60S acidic ribosomal protein P) and analysed by the ΔΔ*Ct* method.

Expression of the following genes was measured: Abca1 (ATP-binding cassette transporter A1), *Actb* (beta actin), *Aif1* (allograft inflammatory factor 1), *Apoe* (apolipoprotein E), *Bdnf* (Brain-derived neurotropic factor), Bmp2 and Bmp4 (Bone morphogenic protein 2 and 4), *Car2* (carbonic anhydrase 2), Ch25h (Cholesterol 25-Hydroxylase ), *Cntf* (ciliary neurotrophic factor), *Cyp27a1* (Sterol 27-hydroxylase ), *Cyp46a1* (Cholesterol 24-hydroxylase), *Cyp51a1* (Sterol 14 alpha-demethylase), *Dhcr24* (24-Dehydrocholesterol reductase), *Egf* (epidermal growth factor), *Fdft1* (Farnesyl-Diphosphate Farnesyltransferase 1), members of the *Fgf* (fibroblast growth factor) gene family, Gfap (glial fibrillary acidic protein), Gmcsf (granulocyte-macrophage colony-stimulating factor, CSF2), *H2-DMb2* (MHCII, major histocompatibility complex class II), *Hmgcr* (3-Hydroxy-3-Methylglutaryl-CoA Reductase), *Hmgcs1* (3-Hydroxy-3-methylgutaryl-CoA synthase 1), *Ifng* (interferon gamma), *Igf1* (insulin-like growth factor 1), *Il2* (interleukin 2), *Il17*, *Inhba* (inhibin beta-A, also called activin beta-A), *Ldlr* (Low density lipoprotein receptor), *Lrp1* (Low density lipoprotein receptor-related protein 1), *Mvk* (Mevalonate kinase), *Ntf3* (Neurotrophin 3), *Ngf* (Nerve growth factor ), *Nr1h3* (Liver X receptor alpha, LXR alpha), *Nr1h2* (Liver X receptor beta, LXR beta), *Olig2* (oligodendrocyte lineage transcription factor 2), *Pdgfa* (platelet derived growth factor alpha), *Plp1* (proteolipid protein 1), Ptn (Pleiotrophin), *Rxrg* (Retinoic X receptor gamma, RXR gamma), *S100b* (S100 calcium-binding protein B), *Shh* (sonic hedgehog), *Srbf2* (sterol regulatory element binding transcription factor 2), *Tnf* (tumour necrosis factor), Vldlr (Very low density lipoprotein receptor). All primer sequences are listed in Supplementary Table 3.

### Cell cultures

For primary oligodendrocyte cultures, dissected cortices of newborn mice or rats were digested in 0.25 mg ml^−1^ Trypsin/EDTA for 10 min followed by triturating and plating in plating media (DMEM 4.5 g l^−1^ Glucose, 10% fetal calf serum containing about 300 μg ml^−1^ cholesterol, GlutaMAX, penicillin/streptomycin). About 14 days after plating, OPCs were isolated by differential shaking and lectin panning, and plated in differentiating Sato media (DMEM 4.5 g l^−1^ glucose, 4 mM glutamine, 5 μg ml^−1^ insulin, 16 μg ml^−1^ putrescine, 6.2 ng ml^−1^ progesterone, 5 ng ml^−1^ sodium selenite, 400 ng ml^−1^ L-thyroxine, 400 ng ml^−1^ triiodothyroxine, 50 μg ml^−1^ holo-transferrin, penicillin/streptomycin, lacking any cholesterol source). Cell purity was routinely determined by immune stainings and always exceeded 95%. Cholesterol (10 μg ml^−1^) was added from a 10 mg ml^−1^ stock solution in ethanol. Control cultures received 0.1% ethanol. In case of growth factor supplementation assays, cells were allowed to adhere for 2 h before treatment with EdU (5-ethynyl-2′-deoxyuridine, 10 μM, Invitrogen) and growth factors (FGF1, FGF2; Peprotech) at 100 ng ml^−1^ for proliferation experiments or 45 ng ml^−1^ FGF1 plus 80 ng ml^−1^ FGF2 or 90 ng ml^−1^ FGF1 plus 35 ng ml^−1^ FGF2 for differentiation experiments. Cultures were fixed with PFA and permeabilized with 0.5% Triton X100.

Myelinating co-cultures were established as described^70^ with minor modifications. Briefly, six E13 embryonic spinal cords per culture were digested in 0.125% Trypsin solution in HBSS (without Ca^+2^ and Mg^+2^) at 37 °C for 20 min. After stopping the digestion with 1 ml plating media (DMEM, 25% horse serum, 25% HBSS, 50 μg ml^−1^ DNAse) the tissue was homogenized by gentle trituration and centrifuged for 5 min. 150,000 cells were plated per poly-L-lysine coated coverslip; 3 coverslips per 35 mm Petri dish. After cell attachment in plating media, differentiation media was added (low glucose DMEM, 10 μg ml^−1^ insulin, 10 ng ml^−1^ biotin, 50 nM hydrocortisone, 0.5% N1-mix). N1 mix was 1 mg ml^−1^ apo-transferrin, 20 mM putrescine, 4 μM progesterone, and 6 μM sodium selenite. 50% media change was performed every 24–48 h with differentiation media. After 12 days, insulin was removed from differentiation media. Coverslips were fixed with PFA after 20, 24 and 28 days in culture and permeabilized with −20 °C methanol for 10 min.

Fixed and permeabilized cells were blocked with 10% horse serum in PBS and incubated with primary antibodies in blocking solution followed by secondary antibodies together with click-it kit for detection of EdU and DAPI for nuclear staining. Coverslips were mounted on slides with aqua polymount. On five randomly chosen visual fields of primary oligodendrocyte cultures (× 10 magnification), stainings were evaluated. For differentiation of oligodendrocytes, CNP and MBP positive cells were categorized according to morphological criteria (see Fig. 7a). New oligodendrocytes (new OL) were CNP-positive MBP-negative cells with complex processes. MBP+OL cells contained few MBP-positive intracellular spots but did not form sheaths. Mature oligodendrocytes were CNP- and MBP-positive with sheaths. In myelinating co-cultures, the axonal (SMI31) area and the area with myelin sheaths (MBP) of seven randomly chosen visual fields of myelinating co-cultures (× 10 magnification) was measured after binarization of thresholded images. Specimens were analysed on an Axiophot observer.Z1 (Zeiss) equipped with an AxioCam MRm and the ZEN 2012 blue edition software and evaluated with Image J software. Microscope settings are listed in Supplementary Table 4.

### Statistical analyses

Statistical evaluation was done by unpaired Student's *t*-test for pairwise comparisons or by ANOVA for comparisons of more than two groups as stated in the figure legends. Two-way ANOVA was combined with a post test to evaluate individual groups. For all statistical tests, significance was measured against an alpha value of 0.05. All error bars show s.e.m. *P* values are shown as *P<0.05; **P<0.01; ***P<0.001. No statistical methods were used to predetermine sample sizes, but our sample sizes are similar to those reported in previous publications^25^,^28^. Data analysis was performed blind to the experimental groups.

### Data availability

All data generated or analysed during this study are included in this published article (and its supplementary information files) or available from the authors on request.

## Additional information

**How to cite this article:** Berghoff, S. A. *et al*. Dietary cholesterol promotes repair of demyelinated lesions in the adult brain. *Nat. Commun.* **8,** 14241 doi: 10.1038/ncomms14241 (2017).

**Publisher's note:** Springer Nature remains neutral with regard to jurisdictional claims in published maps and institutional affiliations.

## Supplementary Material

Supplementary Information Supplementary Figures, Supplementary Tables and Supplementary References
