# Supplementary material for: Dietary cholesterol promotes repair of demyelinated lesions in the adult brain
Source: Nat Commun. 2017 Jan 24;8:14241. doi: 10.1038/ncomms14241 (PMC5286209; doi:10.1038/ncomms14241)
Supplement: Supplementary Information — Supplementary Figures, Supplementary Tables and Supplementary References [file ncomms14241-s1.pdf]

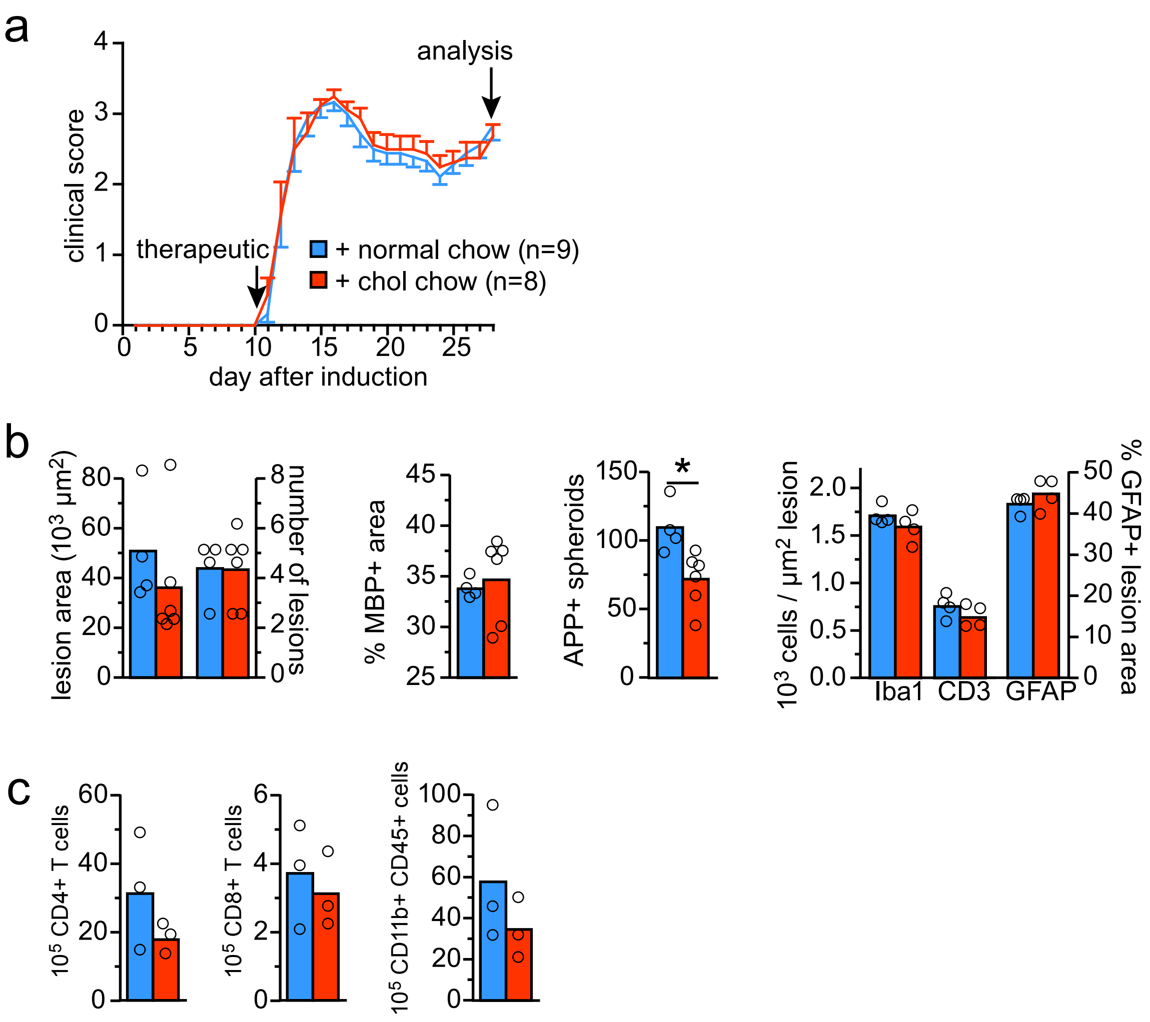

Supplementary Figure 1.

Therapeutically fed cholesterol does not aggravate EAE lesions.

a) Clinical score of mice with EAE, when cholesterol feeding started therapeutically with the onset of symptoms (n=8-9 mice).

b) Histopathological assessment of lumbar spinal cord sections as done in prophylactic cholesterol feeding experiments (Fig. 1). LFB/PAS staining was used to determine the lesion area and number of lesions per section. Immuno-labeling for myelin basic protein was used to determine the percent of myelinated area within a lesion. On sections immuno-labeled for APP, the number of axonal speroids per square mm white matter area was counted, as a readout of axonal damage. Unpaired student's t-test revealed significantly less axonal damage in cholesterol fed animals (*, $P<0.05$). Sections triple stained for microglia / macrophages, T cells, and astrocytes (Iba1-CD3-GFAP triple immuno-labeling) were used to assess the cellular composition of lesions. Bars represent mean values (n=4-6 animals) with individual data points.

c) Absolute number of abTCR/CD4 and abTCR/CD8 T cells and CD45/CD11b myeloid (macrophages/microglia) cells per gram spinal cord was determined by flow cytometry from mice fed normal chow or cholesterol enriched chow (n=3 animals). Bars represent means with individual data points.

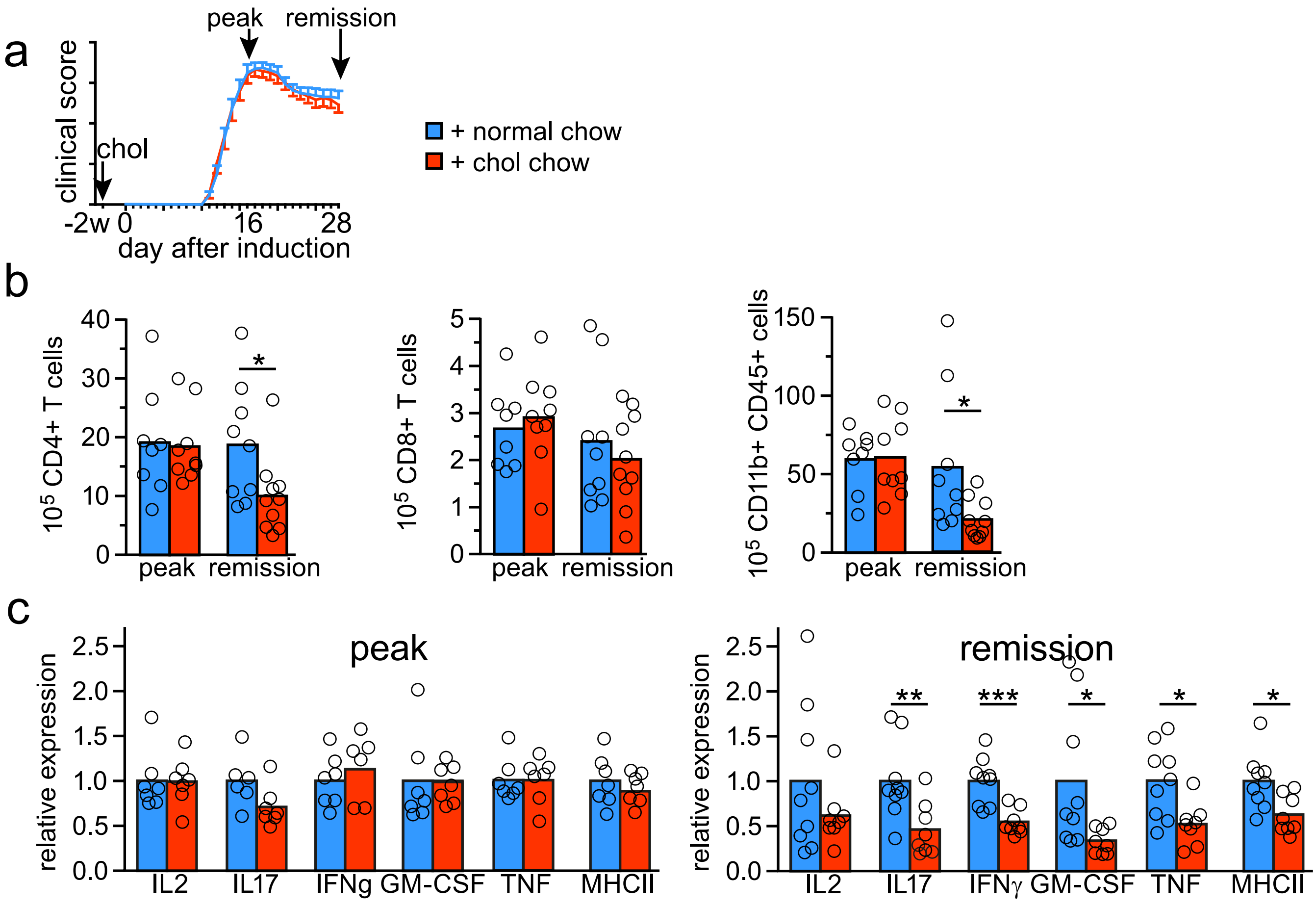

Supplementary Figure 2.
Immune cell infiltration and the inflammatory milieu in EAE.
a) Illustration of time points of analysis at the peak of clinical symptoms (16-18 dpi) and at remission (28 dpi), showing the clinical score of mice with EAE fed normal chow (bue) or cholesterol enriched chow (red).
b) Absolute number of abTCR/CD4 and abTCR/CD8 T cells and CD45/CD11b myeloid (macrophages/microglia) cells per gram spinal cord was determined by flow cytometry from mice fed normal chow or cholesterol enriched chow (n=8-10 animals, 2 independent experiments). Bars represent mean values with individual data points. One-way ANOVA with Tukey's post test revealed significantly less infiltration by CD4 T cells and reduced density of myeloid cells (microglia / macrophages) during remission (*, $P< 0.05$).
c) Quantitative RT-PCR analysis to assess the inflammatory milieu in spinal cord lysates of mice fed normal chow or chow supplemented with cholesterol at peak of clinical symptoms and in remission, determining the expression of IL2, IL17, IFNg, TNF, GM-CSF, and MHC-II. Values were normalized to chow fed mice. Bars represent the means (n=7 animals at peak, n=9 animals in remission) with individual data points. Significance of cholesterol supplementation on expression of individual genes was assessed by student's t tests (*, $P<0.05$; **, $P<0.01$; ***, $P<0.001$).

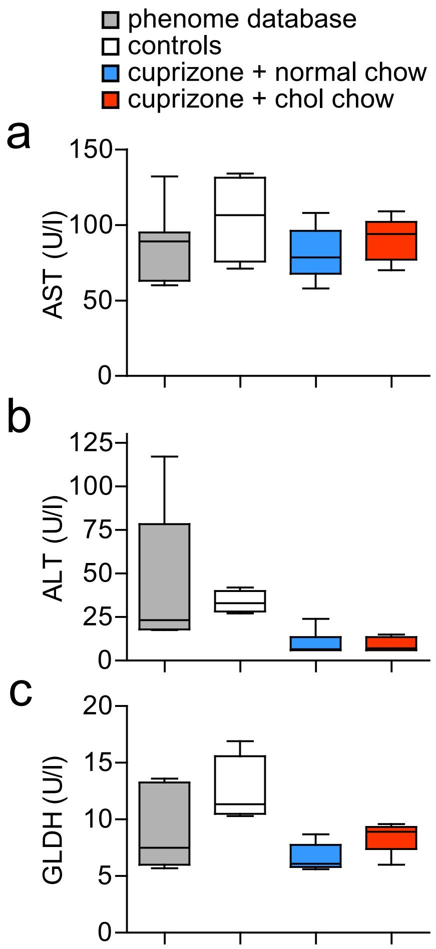

Supplementary Figure 3.
Normal liver function in cuprizone treated animals.
Activity of a) AST (aspartate transaminase), b) ALT (alanine transaminase), and c) GLDH (glutamate dehydrogenase) were determined in serum from mice after 6 weeks cuprizone with or without cholesterol supplementation (n=5-6 animals) and own untreated controls (n=4). Median with 2.5th - 97.5th percentiles were plotted together with published reference intervals (AST n=7, ALT n=5, GLDH n=5; Jackson Laboratory mouse phenome database (Supplementary Refs. 1, 2)).

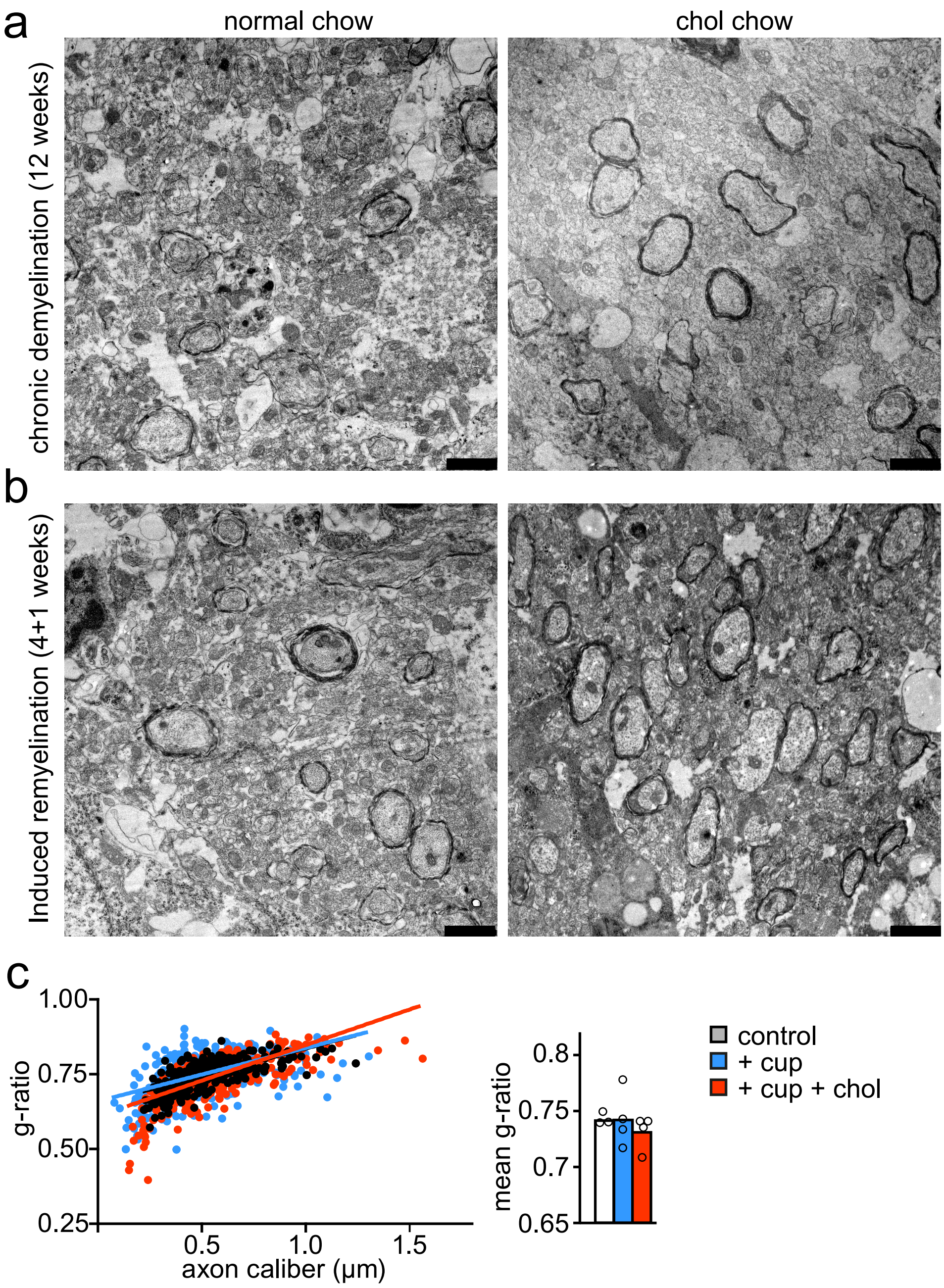

Supplementary Figure 4.
Ultrastructural analysis of cuprizone treated mice.
Representative electron micrographs of the corpus callosum of mice (a) that were chronically treated with cuprizone (12 weeks) with or without cholesterol chow or (b) were treated according to the „induced remyelination" paradigm (demyelination with cuprizone in normal chow for four weeks, followed by remyelination for 1 week with or without cholesterol chow, 4+1). Scale bars, 1 µm.
c) G-ratio analysis and mean g ratio in the corpus callosum of mice treated according to the „induced remyelination" paradigm (4+1) showing individual data points and mean g-ratios of cuprizone treated mice with and without cholesterol supplementation (n=4 animals) and untreated control mice (n=3 animals; 80-100 myelinated axons per animal).

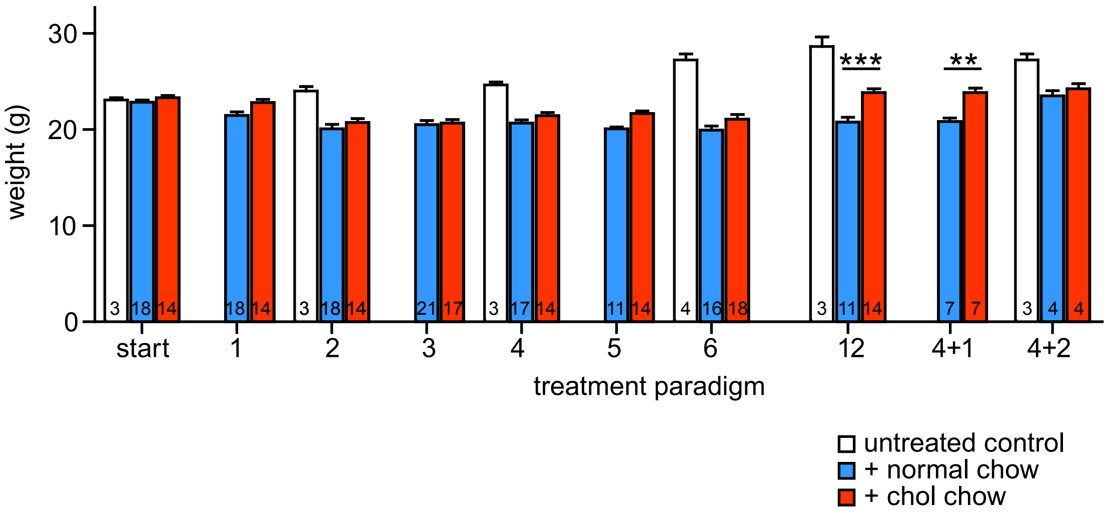

Supplementary Figure 5.
Body weight of cuprizone treated animals.
Body weight of cuprizone treated animals on normal or cholesterol chow, and untreated control mice. Shown are mice at the beginning and from 1 to 12 weeks on cuprizone, and animals treated according to the “induced remyelination” paradigm (4+1 and 4+2, compare Fig. 5a). Number of animals at condition are depicted in the bars. Body weight is expressed as mean ± s.e.m. Two way ANOVA revealed a significant influence of cholesterol on the weight of cuprizone treated animals ($P<0.0001$), and Bonferoni post tests showed a significant difference at the 12 weeks ($P<0.0001$) and 4+1 ($P<0.001$) weeks time points.

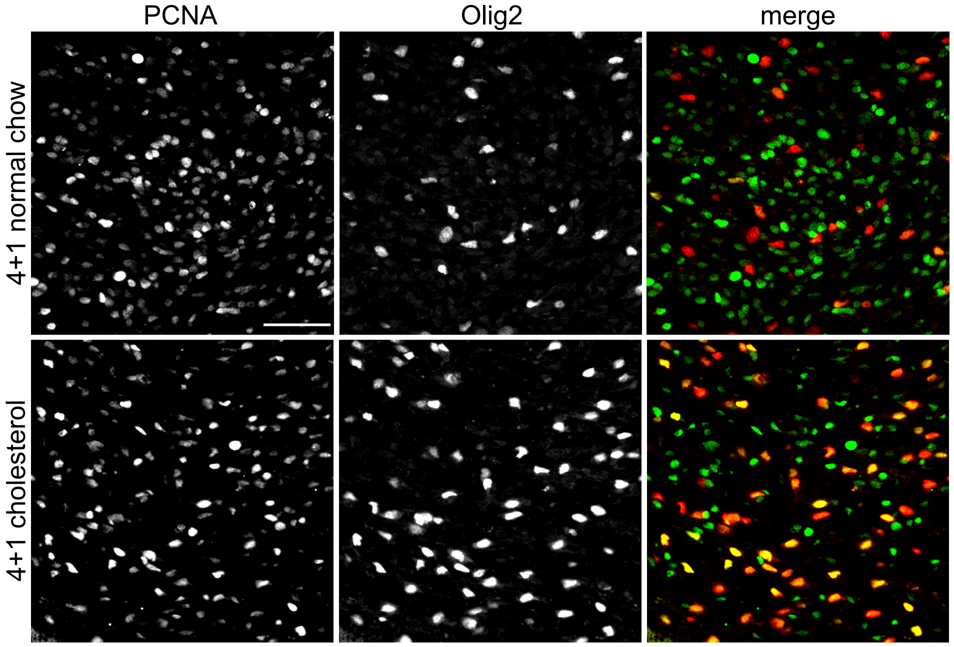

Supplementary Figure 6.
OPC proliferation.
Representative images of proliferating OPCs (PCNA positive, Olig2 positive) in the corpus callosum of mice in the "induced remyelination" paradigm (demyelination for 4 weeks, followed by remyelination for one week, 4+1) in the presence of absence of cholesterol supplementation (scale 50 µm).

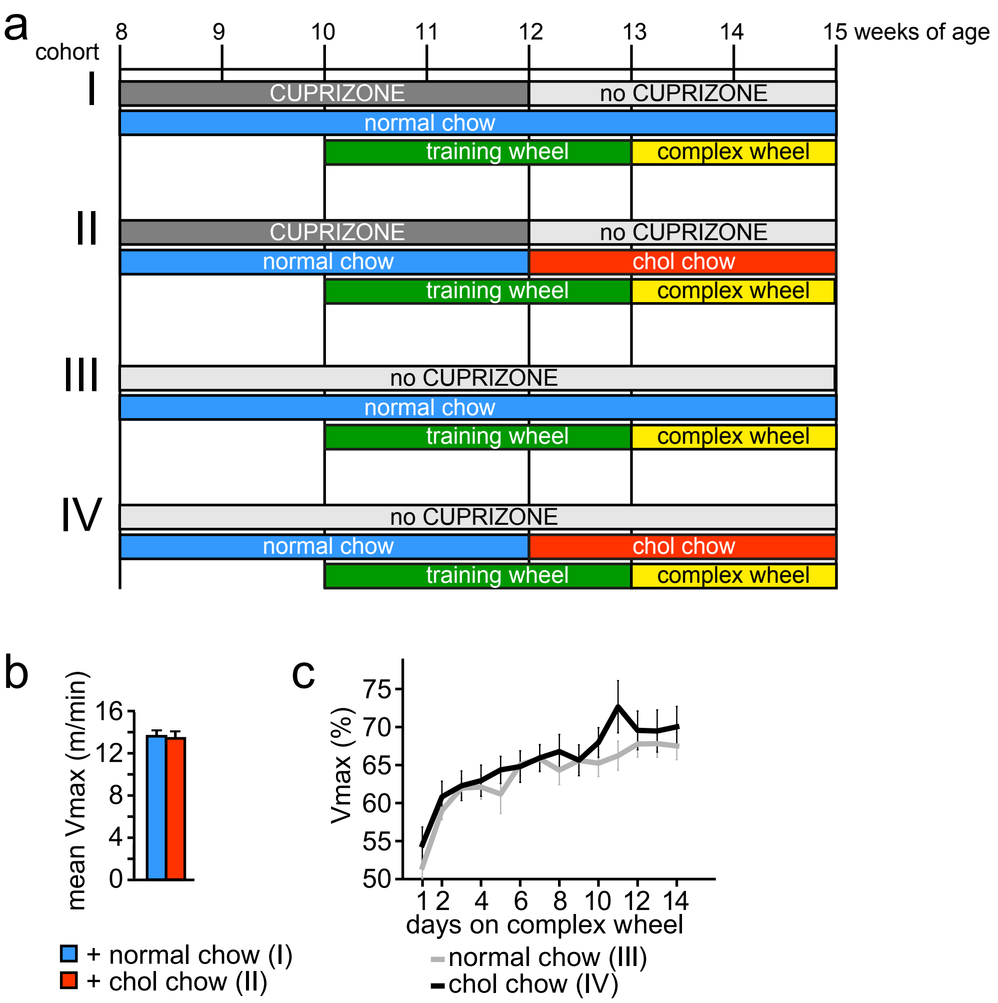

Supplementary Figure 7.
Motor performance on running wheel.
a) Treatment scheme of experimental cohorts. Mice in the "induced remyelination" paradigm (I, II) received cuprizone in normal chow for 4 weeks followed by normal chow with or without added cholesterol. Control groups (III, IV) did not receive cuprizone. A training wheel was placed into cages two weeks after the start of experiment for a duration of three weeks to improve cardiopulmonary and musculoskeletal strength. Mean maximum running speed (Vmax) of the last 7 days on the training wheel was set as reference (100%) for each individual animal. Then a complex wheel replaced the training wheel and percent Vmax for each individual was calculated (control groups III and IV; experimental groups see Fig. 5g) which is a measure for bilateral sensorimotor coordination that likely involves the cerebellum and motor cortex and connecting white matter such as the corpus callosum.
b) Vmax on training wheel is not influenced by dietary cholesterol supplementation. Mean Vmax of each day of the third week on training wheel of cohorts I and II (n=6 animals).
c) Vmax on complex wheel is not influenced by dietary cholesterol in control groups (III, IV) without cuprizone (n=13 animals).

a

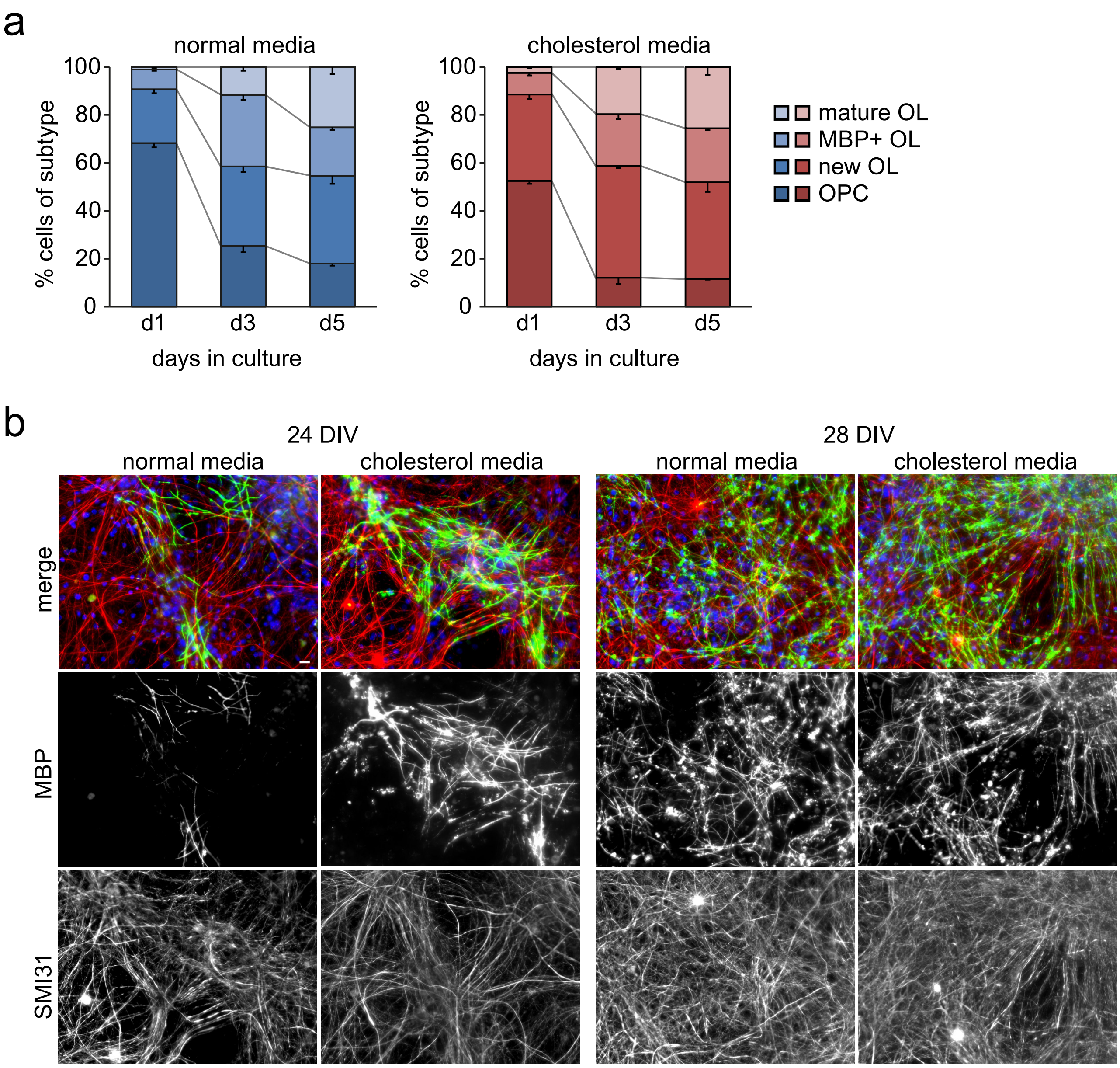

Supplementary Figure 8.
Increased the rate of oligodendrocyte differentiation and myelination in vitro.
a) Primary OPCs were plated in differentiating Sato media with or without 10 µg/ml cholesterol (f.c.). Coverslips were fixed after 1, 3 or 5 days, stained for CNP and MBP and scored as outlined in the methods section.
b) Representative images of myelinating cocultures at 24 and 28 days in vitro (DIV), established from embryonic spinal cord in the presence or absence of cholesterol supplementation and stained for myelin segments (MBP, green), axons (SMI31, red), and nuclei (DAPI, blue) (scale bar, 20 µm).

Supplemental Table 1: Gene expression profile in corpus callosum of mice in "induced remyelination" paradigm

Quantitative RT-PCR on dissected corpus callosi from mice after one week of induced remyelination with or without cholesterol (cuprizone for 4 weeks followed by one week remyelination) and untreated controls. Values are expressed as mean ratio ± s.e.m. of untreated control mice (set to 1). Significance of cholesterol supplementation in cuprizone treated animals was assessed by student's t tests (n=4 animals per group).

Induced remyelination		Fold expression		
**Gene name and function**	**Gene symbol**	**Normal diet**	**Chol diet**	**P value**
**Oligodendrocyte genes**				
Oligodendrocyte lineage transcription factor 2	*Olig2*	0.62 ± 0.22	2.19 ± 0.13	0.00087
Proteolipid protein 1	*Plp1*	0.44 ± 0,05	2.38 ± 0.17	0.00015
Carbonic anhydrase 2	*Car2*	0.20 ± 0.18	0.40 ± 0.14	0.00888
**Glial markers**				
Allograft inflammatory factor 1	*Aif1*	12.46 ± 0.18	4.86 ± 0.24	0.00772
Glial fibrillary acidic protein	*Gfap*	12.29 ± 0.07	15.42 ± 0.05	0.01873
S100 calcium-binding protein beta	*S100b*	1.53 ± 0.18	2.22 ± 0.05	0.04870
**Cholesterol synthesis and regulation**				
3-Hydroxy-3-methylgutaryl-CoA synthase 1	*Hmgcs1*	0.06 ± 0.23	0.20 ± 0.29	0.00817
3-Hydroxy-3-methylgutaryl-CoA reductase1	*Hmgcr*	0.19 ± 0.19	0.38 ± 0.09	0.00715
Mevalonate kinase	*Mvk*	0.41 ± 0.13	0.69 ± 0.06	0.00368
Farnesyl-diphosphate farnesyltransferase 1	*Fdft1*	0.39 ± 0.03	0.67 ± 0.02	0.00087
Sterol 14 alpha-demethylase	*Cyp51a1*	0.19 ± 0.06	0.27± 0.08	0.00465
24-Dehydrocholesterol reductase	*Dhcr24*	0.19 ± 0.35	0.60 ± 0.29	0.02004
Sterol regulatory element binding transcription factor 2	*Srebf2*	0.37 ± 0.14	0.51 ± 0.06	0.04666
**Cholesterol uptake, transport and secretion**				
Apolipoprotein E	*Apoe*	8.73 ± 0.09	7.94 ± 0.09	0.37224
Low density lipoprotein receptor-related protein 1	*Lrp1*	3.56 ± 0.06	6.41 ± 0.12	0.00167
Low density lipoprotein receptor	*Ldlr*	0.31 ± 0.08	0.79 ± 0.14	0.00040
Very low density lipoprotein receptor	*Vldlr*	0.58 ± 0.09	0.87 ± 0.16	0.03860
ATP-binding cassette transporter A1	*Abca1*	29.41 ± 0.14	11.22 ± 0.03	0.00015
Sterol 27-hydroxylase	*Cyp27a1*	1.84 ± 0.36	2.02 ± 0.32	0.81516
Cholesterol 25-Hydroxylase	*Ch25h*	6.20 ± 0.22	7.20 ± 0.37	0.67972
Cholesterol 24-hydroxylase	*Cyp46a1*	0.16 ± 0.35	0.23 ± 0.27	0.30948
Liver X receptor alpha	*Nr1h3*	1.00 ± 0.04	0.96 ± 0.10	0.76459
Liver X receptor beta	*Nr1h2*	1.11 ± 0.10	0.99 ± 0.03	0.37539
Retinoic X receptor gamma	*Rxrg*	0.14 ± 0.03	0.15 ± 0.09	0.95361
**Fibroblast growth factor family**				
Fibroblast growth factor 1	*Fgf1*	0.44 ± 0.16	0.92 ± 0.13	0.00427
Fibroblast growth factor 2	*Fgf2*	7.67 ± 0.09	3.12 ± 0.11	0.00021
Fibroblast growth factor 3	*Fgf3*	0.03 ± 0.79	0.67 ± 0.54	0.00695
Fibroblast growth factor 7	*Fgf7*	9.96 ± 0.12	5.23 ± 0.15	0.00589
Fibroblast growth factor 8	*Fgf8*	0.95 ± 0.17	3.17 ± 0.38	0.00965
Fibroblast growth factor 9	*Fgf9*	0.14 ± 0.17	0.44 ± 0.28	0.00447
Fibroblast growth factor 12	*Fgf12*	0.20 ± 0.21	0.52 ± 0.35	0.02539
Fibroblast growth factor 17	*Fgf17*	0.90 ± .24	1.79 ± 0.13	0.02323
Fibroblast growth factor 22	*Fgf22*	1.44 ± 0.27	5.27 ± 0.51	0.03037
**Other growth factors**				
Brain-derived neurotropic factor	*Bdnf*	0.16 ± 0.16	0.19 ± 0.28	0.53091
Ciliary neurotrophic factor	*Cntf*	20.77 ± 0.24	19.54 ± 0.39	0.87452
Epidermal growth factor	*Egf*	7.11 ± 0.16	6.56 ± 0.21	0.71377
Insulin like growth factor 1	*Igf1*	11.72 ± 0.11	13.88 ± 0.18	0.35575
Nerve growth factor	*Ngf*	2.00 ± 0.23	2.00 ± 0.20	0.94831
Neurotrophin 3	*Ntf3*	1.35 ± 0.21	1.23 ± 0.42	0.82436
Platelet derived growth factor alpha	*Pdgfa*	12.59 ± 0.04	8.44 ± 0.02	0.00003
Bone morphogenic protein 2	*Bmp2*	1.35 ± 0.21	2.18 ± 0.10	0.04285
Bone morphogenic protein 4	*Bmp4*	8.34 ± 0.18	7.55 ± 0.20	0.61971
Sonic hedgehog	*Shh*	0.24 ± 0.22	0.39 ± 0.08	0.03463
Inhibin beta-A	*Inhba*	2.49 ± 0.14	2.31 ± 0.33	0.80868
Pleiotrophin	*Ptn*	4.44 ± 0.19	4.93 ± 0.25	0.64768

Supplemental Table 2: Gene expression profile in corpus callosum of mice in "chronic demyelination" paradigm

Quantitative RT-PCR on dissected corpus callosi from chronically demyelinated mice with or without cholesterol (cuprizone for 12 weeks) and untreated controls. Values are expressed as mean ratio ± s.e.m. of untreated control mice (set to 1). Significance of cholesterol supplementation in cuprizone treated animals was assessed by student's t tests (n=4 animals per group).

Chronic cuprizone		Fold expression		
**Gene name and function**	**Gene symbol**	**Normal diet**	**Chol diet**	**P value**
**Oligodendrocyte genes**				
Oligodendrocyte lineage transcription factor 2	*Olig2*	0.24 ± 0.06	0.38 ± 0.03	0.00000
Proteolipid protein 1	*Plp1*	0.10 ± 0.10	0.19 ± 0.11	0.00120
Carbonic anhydrase 2	*Car2*	0.16 ± 0.03	0.32 ± 0.14	0.00417
**Glial markers**				
Allograft inflammatory factor 1	*Aif1*	2.49 ± 0.18	2.24 ± 0.28	0.45346
Glial fibrillary acidic protein	*Gfap*	6.67 ± 0.08	4.75 ± 0.04	0.00104
S100 calcium-binding protein beta	*S100b*	1.96 ± 0.05	1.47 ± 0.11	0.00870
**Cholesterol synthesis and regulation**				
3-Hydroxy-3-methylgutaryl-CoA synthase 1	*Hmgcs1*	0.32 ± 0.13	0.33 ± 0.03	0.84110
3-Hydroxy-3-methylgutaryl-CoA reductase1	*Hmgcr*	0.73 ± 0.02	0.59 ± 0.08	0.00404
Mevalonate kinase	*Mvk*	0.96 ± 0.02	0.70 ± 0.12	0.00281
Farnesyl-diphosphate farnesyltransferase 1	*Fdft1*	0.87 ± 0.02	0.72 ± 0.05	0.00870
Sterol 14 alpha-demethylase	*Cyp51a1*	0.42 ± 0.20	0.48 ± 0.07	0.53237
24-Dehydrocholesterol reductase	*Dhcr24*	0.51 ± 0.16	0.57 ± 0.06	0.55446
Sterol regulatory element binding transcription factor 2	*Srebf2*	0.88 ±0.06	0.72 ± 0.04	0.00700
**Cholesterol uptake, transport and secretion**				
Apolipoprotein E	*Apoe*	2.04 ± 0.05	1.52 ± 0.11	0.00834
Low density lipoprotein receptor-related protein 1	*Lrp1*	3.30 ± 0.05	2.25 ± 0.11	0.00133
Low density lipoprotein receptor	*Ldlr*	0.83 ± 0.03	0.60 ±0.09	0.00080
Very low density lipoprotein receptor	*Vldlr*	2.14 ± 0.12	1.68 ± 0.13	0.39744
ATP-binding cassette transporter A1	*Abca1*	5.17 ± 0.09	3.73 ± 0.11	0.00920
Sterol 27-hydroxylase	*Cyp27a1*	0.26 ± 0.16	0.25 ± 0.03	0.82481
Cholesterol 25-Hydroxylase	*Ch25h*	1.57 ± 0.22	1.50 ± 0.25	0.85498
Cholesterol 24-hydroxylase	*Cyp46a1*	0.72 ± 0.21	0.64 ± 0.09	0.58260
Liver X receptor alpha	*Nr1h3*	0.51 ± 0.02	0.55 ± 0.05	0.59219
Liver X receptor beta	*Nr1h2*	0.98 ± 0.07	0.79 ± 0.07	0.12759
Retinoic X receptor gamma	*Rxrg*	1.18 ± 0.07	1.08 ± 0.13	0.56939
**Fibroblast growth factor family**				
Fibroblast growth factor 1	*Fgf1*	0.54 ± 0.01	1.02 ± 0.07	0.00002
Fibroblast growth factor 2	*Fgf2*	4.32 ± 0.01	2.70 ± 0.06	0.00057
Fibroblast growth factor 3	*Fgf3*	0.79 ± 0.10	1.58 ± 0.09	0.00099
Fibroblast growth factor 7	*Fgf7*	3.31 ± 0.52	3.76 ± 0.52	0.33069
Fibroblast growth factor 8	*Fgf8*	0.39 ± 0.27	0.51 ± 0.16	0.31460
Fibroblast growth factor 9	*Fgf9*	0.59 ± 0.10	0.60 ± 0.06	0.88087
Fibroblast growth factor 12	*Fgf12*	0.47 ± 0.08	0.42 ± 0.16	0.51466
Fibroblast growth factor 17	*Fgf17*	1.51 ± 0.06	2.30 ± 0.02	0.00000
Fibroblast growth factor 22	*Fgf22*	3.57 ± 0.15	3.62 ± 0.19	0.94283
**Other growth factors**				
Brain-derived neurotropic factor	*Bdnf*	1.82 ± 0.25	2.45 ± 0.65	0.17036
Ciliary neurotrophic factor	*Cntf*	5.32 ± 0.09	3.86 ± 0.17	0.02809
Epidermal growth factor	*Egf*	1.93 ± 0.14	2.51 ± 0.08	0.02271
Insulin like growth factor 1	*Igf1*	2.79 ± 0.03	2.49 ± 0.08	0.01711
Nerve growth factor	*Ngf*	0.39 ± 0.09	0.44 ± 0.15	0.48108
Neurotrophin 3	*Ntf3*	0.61 ± 0.17	0.76 ± 0.19	0.20164
Platelet derived growth factor alpha	*Pdgfa*	8.83 ± 0.11	4.70 ± 0.19	0.00305
Bone morphogenic protein 2	*Bmp2*	1.01 ± 0.25	0.99 ± 0.19	0.92774
Bone morphogenic protein 4	*Bmp4*	1.15 ± 0.03	1.73 ± 0.20	0.00429
Sonic hedgehog	*Shh*	0.67 ± 0.08	0.58 ± 0.27	0.45346
Inhibin beta-A	*Inhba*	1.33 ± 0.11	1.29 ± 0.08	0.78641
Pleiotrophin	*Ptn*	1.89 ± 0.08	1.78 ± 0.18	0.68471

Supplementary Table 3: List of primer sequences

All primers used for expression analysis were intron-spanning (5'-3'; forward - reverse).

Rplp0	GATGCCCAGGGAAGACAG – ACAATGAAGCATTTTGGATAATCA
Hprt1	TCCTCCTCAGACCGCTTTT – CCTGGTTCATCATCGCTAATC
Plp1	TCAGTCTATTGCCTTCCCTAGC – AGCATTCCATGGGAGAACAC
Car2	CAAGCACAACGGACCAGA – ATGAGCAGAGGCTGTAGG
Olig2	AGACCGAGCCAACACCAG – AAGCTCTCGAATGATCCTTCTTT
Aif1	TGTTTTTCTCCTCATACATCAGAATC – CCGAGGAGACGTTCAGCTAC
Gfap	TGCTCCTGCTTCGAGTCCTT – CAAGAGGAACATCGTGGTAAAGA
S100b	AACAACGAGCTCTCTCACTTCC – CTCCATCACTTTGTCCACCA
Hmgcs1	GGGTCTGATCCCCTTTGG – ACGATTCCCACATCTTTTGG
Hmgcr	TGATTGGAGTTGGCACCAT – TGGCCAACACTGACATGC
Mvk	CTCAAGGACGGGGTCTCC – GGCCCACTTGTTGATTGACT
Fdft1	TCAATCAGACCAGTCGCAGC – GTGCCGTATGTCCCCATCC
Srebf2	ACCTAGACCTCGCCAAAGGT – GCACGGATAAGCAGGTTTGT
Apoe	GACCCTGGAGGCTAAGGACT – AGAGCCTTCATCTTCGCAAT
Cyp51a1	TTAGAACAGAAAGCAGTGTGTGG –TGCATCTATCAAGTAAATTCAGATCC
Dhcr24	GGTCATGACGGACGACGTA – AGGGCTTGTAGTAACTGCCAAT
Lrp1	ACCACCATCGTGGAAAATG – GTCCCAGCCACGGTGATA
Ldlr	TTCCTGTCCATCTTCTTCCCTA – CAAAGTTTATGCTGTTGATTGTGA
Vldlr	AAGTCAGTGTTCCCCCAAAA – TGCTGCCATCACTAAGAGCA
Abca1	CTGTTTCCCCCAACTTCTG – TCTGCTCCATCTCTGCTTTC
Cyp27a1	ATGGGATCTTCATCGCACA – CGTTTAAGGCATCCGTGTAGA
Ch25h	TGCTACAACGGTTCGGAGC – AGAAGCCCACGTAAGTGATGAT
Cyp46a1	AACTTTGTCACCTTCTTCATTGC – CCATCACTGTGAATGCCAGA
Fgf1	GGACACCGAAGGGCTTTTAT – GCATGCTTCTTGGAGGTGTAA,
Fgf2	CCAACCGGTACCTTGCTATG – GATTCCAGTCGTTCAAAGAAGAA,
Fgf3	TGAGAACAGCGCCTATAGCA – GTACCGCCCAGAAAAGAGC,
Fgf7	GGCAATCAAAGGGGTGGA – CCTCCGCTGTGTGTCCATTTA,
Fgf8	GCTGAGCTGCCTGCTGTT – AGCTCGGAGCAGGGAAGT,
Fgf9	GGGGAGCTGTATGGATCAGA – TCCCGTCCTTATTTAATGCAA
Fgf12	CAAGGACGAAAACAGCGACT – CTCCATTCATGGCCACATAA
Fgf17	TATGAACAAGAGGGGCAAGC – CTCGGTGAACACGCAGTCT
Fgf22	CCAGGACAGTATAGTGGAGATCC – AGTAGACCCGCGACCCATAG
Pdgfa	CTCTTGGAGATAGACTCCGTAG – ACTTCTCTTCCTGCGAATGG
Cntf	GACCTGACTGCTCTTATGGAATCT – GCCTGGAGGTTCTCTTGGA
Igf1	AGCAGCCTTCCAACTCAATTAT – GAAGACGACATGATGTGTATCTTTATC
Bdnf	GCATCTGTTGGGGAGACAAG – TGGTCATCACTCTTCTCACCTG
Egf	TTGTGACACCTGGAAAACTGA – GTTTGAGAAGTTCGGGGTCA
Ngf	TATACTGGCCGCAGTGAGGT – GGACATTGCTATCTGTGTACGG
Ntf3	GCCAGGCCGGTCAAAAAC – GACAAGGCACACACACAG
Bmp2	CGGACTGCGGTCTCCTAA – GGGGAAGCAGCAACACTAGA
Bmp4	GAGGAGTTTCCATCACGAAGA – GCTCTGCCGAGGAGATCA
Shh	TCCACTGTTCTGTGAAAGCAG – GGGACGTAAGTCCTTCACCA
Inhba	GGGAGTGATCCCTGGAAAC – TCCTCTTCATGGTATTGGCACT
Ptn	CCTCAAGCGGAGTCAAAGAA – CTTTTCCTGGTCCACAGACG
Actb	GTACAACCTCCTTGCAGC TCCT – TTG TCG ACGACGAGCGC
Actb probe	CGCCACCAGTTCGCCATGGAT
Ifng	TCAAGTGGCATAGATGTGGAA GAA –TGGCTCTGCAGGATTTTC ATG
Ifng probe	TCA CCATCCTTT TGC CAGTTCCTCCAG
IL17	ACT TTC AGG GTC GAG AAG ATG CT–TTC TGA ATC TGC CTC TGA ATC CAC
IL17 probe	TGGGTGTGGGCTGCACCTGC
Il2	CAGGATGCTCACCTTCAA ATT TT – CGCAGAGGTCCAAGTTCATCT
Il2 probe	CTTGCCCAAGCAGGCCACAGA ATTG
Gmcsf	GGGCGCCTTGAACATGAC – CGCATAGGTGGTAAC TTGTGT TTC
Gmcsf probe	CCCCCCAACTCCGGAAACGGA
Tnf	TCGAGTGACAAGCCCGTAGC – CTCAGCCACTCCAGCTCC TC
Tnf probe	CGTCGTAGCAAACCACCAAGC AGA
H2-DMb2 (MHCII)	AGCCTTATTCATCGCTTG CAA – AGATGGCGCTCTCGTTCTGT
H2-DMb2probe	ACGGGCTTCAGG ACTGTGCCA CC

Supplementary Table 4: Microscope settings

In all recorded images, LUT was linear covering the full range of data. All experiments were done at 20°C, the depth of all pictures was 8 bit.

Figure #	Staining	Fluorochrome/ Chromophore	Ex/Em (nm)	Filters /Pinhole		Beam-splitter	Exposure time (ms) / Scan speed (Hz)	xyzt dimension (µm)	Pixel dimension (px / µm)
				Excitation	Emission				
Fig.1a		Evans Blue	630 / 690	640 ± 15	690 ± 25	660	100	X: 1392 / Y: 1040	0.98
Fig. 1c		Bodipy-cholesterol	495 / 507	0.000073 m; 1.000275 airy		-	400	X: 1024 / Y: 1024	1.365
Figs.<br>2b, 3b ,4b	CalI	DAPI<br>Alexa 555	358 / 461<br>555 / 580	360 ± 25<br>585 ± 25	445 ± 25<br>605 ± 35	395<br>570	320<br>1200	X: 895.26 / Y: 670.80	1.55
Figs.<br>2b, 3b ,4b	Olig2	DAPI<br>Alexa 555	358 / 461<br>555 / 580	360 ± 25<br>585 ± 25	445 ± 25<br>605 ± 35	395<br>570	100<br>2000	X: 895.26 / Y: 670.80	1.55
S. Fig .2	MBP	DAPI<br>Alexa 555	358 / 461<br>555 / 580	360 ± 25<br>585 ± 25	445 ± 25<br>605 ± 35	395<br>570	150<br>750	X: 2570 / Y: 1950	3.09
S. Fig. 2b	CD3<br>Iba1<br>GFAP	DAPI<br>Alex 488<br>Alex 555<br>Alex 633	358 / 461<br>490 / 525<br>555 / 580<br>638 / 658	360 ± 25<br>500 ± 10<br>585 ± 25<br>640 ± 15	445 ± 25<br>535 ± 15<br>605 ± 35<br>690 ± 25	395<br>515<br>570<br>660	100<br>1100<br>900<br>2100	X: 2130 / Y: 2220	3.09
S. Fig. 7	PCNA<br>TCF 4	DAPI<br>Alexa 488<br>Alex 555	358 / 461<br>490 / 525<br>555 / 580	360 ± 25<br>500 ± 10<br>585 ± 25	445 ± 25<br>535 ± 15<br>605 ± 35	395<br>515<br>570	340<br>1100<br>1400	X: 447.63 / Y: 335.88	3.09
S Fig. 8a	Olig2	DAPI<br>Alexa 555	358 / 461<br>555 / 580	360 ± 25<br>585 ± 25	445 ± 25<br>605 ± 35	395<br>570	550<br>570	X: 798.19 / Y: 861.72	3.09
S. Fig. 8a	MBP	DAPI<br>Alexa 488	358 / 461<br>490 / 525	360 ± 25<br>500 ± 10	445 ± 25<br>535 ± 15	395<br>515	100<br>4000	X: 895.26 / Y: 670.80	1.55
S. Fig. 10	MBP<br>SMI31	DAPI<br>Alex 488<br>Alex 555	358 / 461<br>490 / 525<br>555 / 580	360 ± 25<br>500 ± 10<br>585 ± 25	445 ± 25<br>535 ± 15<br>605 ± 35	395<br>515<br>570	1300<br>3200<br>3200	X: 447.63 / Y: 335.88	3.09
Figs.<br>2b, 3b, 4b	Gallyas	Silver particle	-	-	-	-	18	X: 1420 / Y: 1060	0.98
Figs.<br>2b, 3b, 4b	MAC3	DAB /<br>Hematoxylin	-	-	-	-	16	X: 1420 / Y: 1060	0.98
Figs.<br>2b, 3b, 4b	GFAP	DAB /<br>Hematoxylin	-	-	-	-	15	X: 1420 / Y: 1060	0.98
S. Fig. 2b	LFB-PAS	Luxol fast blue<br>Periodic acid	-	-	-	-	8	X: 2040 / Y:2470	1.95
S. Fig. 2b	APP	DAB /<br>Hematoxylin	-	-	-	-	6.8	X: 2670 / Y:2470	1.95

Supplementary References

1. Bradford, B.U., et al. Interstrain differences in the liver effects of trichloroethylene in a multistrain panel of inbred mice. Toxicol Sci 120, 206-217 (2011).

2. Shockley K, Paigen B, Churchill GA. Diet effects on blood chemistry and lipids in 10 inbred strains of mice. Mouse Phenome Database web site, The Jackson Laboratory, Bar Harbor, Maine USA. http://phenome.jax.org
